# Hepeliviruses in two waterbodies in Berlin, Germany

**DOI:** 10.1007/s00705-022-05688-0

**Published:** 2022-12-25

**Authors:** Roland Zell, Marco Groth, Lukas Selinka, Hans-Christoph Selinka

**Affiliations:** 1 Section of Experimental Virology, Institute for Medical Microbiology, Jena University Hospital, Friedrich Schiller University, Jena, Germany; 2 CF DNA Sequencing, Leibniz Institute on Aging, Fritz Lipmann Institute, Jena, Germany; 3 Section II 1.4 Microbiological Risks, Department of Environmental Hygiene, German Environment Agency, Berlin, Germany

## Abstract

The order *Hepelivirales* comprises RNA viruses of four families (*Alphatetraviridae*, *Benyviridae*, *Hepeviridae,* and *Matonaviridae*). Sequencing of virus genomes from water samples from the Havel River and the Teltow Canal (Teltowkanal) in Berlin, Germany, revealed 25 almost complete and 68 partial genomes of viruses presumably belonging to the order *Hepelivirales*. Only one of these viruses exhibited a relationship to a known member of this order. The members of one virus clade have a polymerase with a permuted order of the conserved palm subdomain motifs resembling the polymerases of permutotetraviruses and birnaviruses. Overall, our study further demonstrates the diversity of hepeliviruses and indicates the enzootic prevalence of hepeliviruses in unknown hosts.

**Supplementary Information:**

The online version contains supplementary material available at 10.1007/s00705-022-05688-0.

The development of next-generation sequencing techniques has enabled analysis of unculturable viruses from sources as diverse as water, soil, and samples of prokaryotic and eukaryotic organisms. As a result, sequence data for thousands of viruses have been obtained, but an immense amount of “viral dark matter” [14] remains to be investigated. Among the many virus groups that are now better understood because of the increase in sequence data are hepeviruses and related viruses of the order *Hepelivirales*. The present classification of the higher-order taxa of RNA viruses is based essentially on the similarity of the RNA-dependent RNA polymerase (RdRp), the only universal gene of all RNA viruses. According to Wolf and coworkers, the global RdRp phylogenetic tree consists of five major branches, with the members of the order *Hepelivirales* belonging to the "alphavirus supergroup" of branch 3 [19]. Except for the similarity of their RdRp, the members of the *Hepelivirales* share few commonalities regarding genome structure, capsid symmetry, or host range: Mono-, bi-, and multipartite viruses have been described. The lengths of genomic RNAs (gRNAs) vary from 6.7 kb to 9.8 kb for viruses with monopartite genomes and up to 16.8 kb in total for the multipartite benyviruses. gRNAs may be polyadenylated (*Benyviridae*, *Hepeviridae*, *Matonaviridae*) or not (*Alphatetraviridae*) and encode a large nonstructural polyprotein at their 5' end with an N-terminal N7-methyltransferase, a central helicase, and a C-terminal 'alpha-like' RdRp. A capsid protein (CP) is encoded either by a subgenomic mRNA (sgRNA) or by a second gRNA molecule (*Benyviridae* and Helicoverpa armigera stunt virus of the family *Alphatetraviridae*). Particles are either icosahedral, with a T = 3 (*Hepeviridae*) or T = 4 lattice (*Alphatetraviridae*, *Matonaviridae*), or rod-like with helical nucleocapsids (*Benyviridae*). The CP of icosahedral viruses has a jelly-roll fold. Only members of the family *Matonaviridae* have enveloped virions. The wide host range includes vertebrates (*Hepeviridae*, *Matonaviridae*), lepidopteran insects (*Alphatetraviridae*), and plants (*Benyviridae*).

In addition to the classified members of the order *Hepelivirales*, a great number of distantly related, unclassified hepe-like viruses (hepeliviruses) have been described. Among these viruses are the bastroviruses from humans, other mammals, and fish [1, 10, 17] as well as so-called ‘bastro-like’ viruses and many other hepe-like viruses detected in mammals, lower vertebrates, invertebrates, plants, sewage, and environmental water samples, e.g., [4, 5, 12, 13, 15–18, 20]. The genomes of these viruses vary from 5 to 12.5 kb in size and contain 2-8 open reading frames (ORFs) indicating that the 'hepe-like' viruses likely comprise several new virus families.

Considering the information given above on viruses in the order *Hepelivirales* and their reservoirs, especially in sewage and environmental water samples, the aim of this project was to analyse the virome of two waterbodies in Berlin, Germany, to reveal the spectrum of viruses and to characterise the viruses detected by phylogenetic analysis. The present study describes 25 almost complete and 68 partial hepelivirus genomes. These viruses were designated Havel hepe-like virus (HHLV) 1 to 38 and Teltowkanal hepe-like virus (TkHLV) 1 to 46. Further, we describe the partial genomes of six astro-like viruses, named Havel astro-like virus (HALV) 1 to 3, and Teltowkanal astro-like virus (TkALV) 1 to 3. None of the viruses belong to a known family of the order *Hepelivirales* or *Stellavirales* except for TkHLV-14. Whereas TkHLV-14 has the P70-like CP of omegatetraviruses, the genome sequences of the remaining viruses display similarity to hepe-like and astro-like viruses from crustacea, aquatic arthropods, and unspecified lophotrochozoa, which is compatible with their detection in environmental water samples.

The methods of sample collection, virus enrichment, RNA preparation, and sequencing have been described previously [21]. Briefly, 50-L freshwater samples were collected from the Teltow Canal (Teltowkanal) and the Havel River in Berlin, Germany (for sampling dates and sampling site coordinates, see Table 1). Virus particles were concentrated by glass wool filtration and eluted from the column with buffer containing 3% beef extract and 50 mM glycine, pH 9.5. After adjusting its pH to 7, the eluate (180 ml) was filtered (0.45 µm). Virus particles were sedimented by ultracentrifugation (100,000 × *g* for 2.5 h at 4°C). Single-end and paired-end sequencing, respectively, was done using a HiSeq 2500 System (Table 1). Sequence data were extracted in FastQ format employing the bcl2fastq tool of Illumina, followed by adapter and quality trimming with Cutadapt (parameters: -q 10 -m 30 -a AGATCGGAAGAGCACACGTCTGAACTCCAGTCA -A AGATCGGAAGAGCGTCGTGTAGGGAAAGAGTGT; -A only with PE data) and removal of amplification duplicons. Assembly was performed with the clc_assembler (parameters: -p fb ss 0850) and metaSPAdes using standard parameters (only for PE data; for parameters and detailed information, see Table 1). A protein database created with all NCBI GenBank entries for the Taxonomy ID 10239 (search term “viruses[organism]”) was screened with DIAMOND [3] and BLAST+ v2.6.0 to identify hepelivirus candidates. The final genome sequences were curated by manual assembly of overlapping contigs if necessary. Sequence alignments were made in MEGA version X [8] and adjusted manually. Codon-adjusted nucleotide sequence alignments were used for maximum-likelihood tree inference with IQ-TREE 2.1.3 for Windows [9]. Optimal substitution models were selected using ModelFinder, implemented in IQ-TREE. Branch support was assessed with 50,000 ultrafast bootstrap replications using UFBoot2, also implemented in IQ-TREE [7].

**Table 1:** Sample information

Sample ID	MR137-16	20161205	MR233-17	MR644-18
Waterbody	Teltow Canal	Teltow Canal	Teltow Canal	Havel River
Sampling site	Berlin, Bäkebrücke	Berlin, Bäkebrücke	Berlin, Bäkebrücke	Berlin, Heerstraße
Sampling site coordinates	52°26′03″N 13°18′57″E	52°26′03″N 13°18′57″E	52°26′03″N 13°18′57″E	52°30′46″N 13°12′14″E
Sampling date	13 July 2016	17 Aug 2016	18 July 2017	28 June 2018
Sample size	50 L	50 L	50 L	50 L
RNA preparation kit	NucliSENS miniMAG (Biomérieux)	NucliSENS miniMAG (Biomérieux)	QIAamp Viral RNA mini kit (QIAGEN)	QIAamp Viral RNA mini kit (QIAGEN)
Sequencing method	HiSeq 2500 50 bp, **SE**, HT	HiSeq 2500 100 bp, **SE**, RR	HiSeq 2500 150 bp, **PE**, RR	HiSeq 2500 150 bp, **PE**, RR
Reads	62,220,180*	55,164,912*	70,018,635^§^	51,902,006^§^
Assembly method	clc/-	clc/-	clc/metaSPAdes	clc/metaSPAdes
Contigs/Scaffolds (>200 nt)	7,952/-	178,946/-	537,529/1,314,849	162,082/388,367
Contigs assigned by DIAMOND to				
Viruses	867	17,816	41,408/66,371	8,810/16,922
*Hepelivirales*	7	18	91/98	34/50
*Stellavirales*	7	26	113/159	76/96

Abbreviations: SE, single-end sequencing; PE, paired-end sequencing; HT, HiSeq 2500 high-throughput mode; RR, HiSeq 2500 Rapid Run mode; clc, clc assembler v5.2.1; metaSPAdes, metaSPAdes assembler v3.15.3 (Python: v3.9.11)

*SE reads after adapter trimming

^§^PE read pairs after adapter trimming and removal of duplicons

Illumina sequencing results varied depending on the yields of virus enrichment and the sequencing method (Table 1). DIAMOND analysis suggested the presence of many scaffolds/contigs belonging to the orders *Hepelivirales* and *Stellavirales*. However, the vast majority of scaffolds/contigs with hepe-like sequences were misassigned to the order *Stellavirales*, and close to 100% of all assignments to lower ranks (family, genus, species) could not be verified by BLAST. Using BLAST, 93 partial or full-length genome sequences of hepe-like viruses were identified, with sizes ranging from 970 nt to 9268 nt, plus six partial astro-like virus genome sequences with lengths from 539 nt to 6815 nt (GenBank accession nos. OP699055 to OP699153; Supplementary Table S1). The genome layout of 21 (almost) complete hepe-like virus genomes is presented in Supplementary Figure S1. Interestingly, many viruses were present in two or more samples with almost identical sequences, indicating that a very similar hepeli virome is present in both waterbodies and in three consecutive years (Supplementary Table S1). Most of the contigs smaller 1 kb were not further characterized.

As a large nonstructural polyprotein with methyltransferase, helicase, and RdRp domains is a common feature of hepeliviruses, phylogenetic analysis of these three domains was conducted. The sequences of 57 of the 93 novel hepeliviruses were suitable for these analyses and included. The sequence alignments contained reference sequences of members of the four *Hepelivirales* families, bastroviruses, and numerous unclassified hepe-like viruses. In addition, the RdRp tree contained representative sequences of mamastroviruses, avastroviruses, and unclassified astro-like viruses as well as the HALV and TkALV sequences (Fig. 1 and Supplementary Fig. S2).

**Fig. 1:**
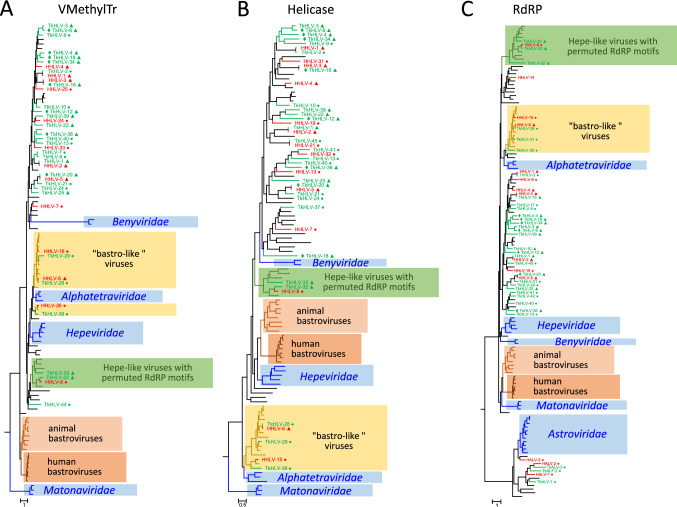
Phylogenetic analysis of the viral methyltransferase (**A**), helicase (**B**), and RdRp domains (**C**) of hepeliviruses. Blue boxes indicate reference virus families; yellow, brown, and green boxes denote unclassified bastroviruses, 'bastro-like' viruses, and hepe-like viruses with permuted RdRp. Sequences of Havel hepe-like viruses (HHLV; printed in red), Teltowkanal hepe-like viruses (TkHLV; printed in green), unclassified hepe-like viruses (printed in black) and reference strains of the families *Alphatetraviridae*, *Benyviridae*, *Hepeviridae*, *Matonaviridae*, and *Astroviridae* (printed in blue) were aligned in MEGA and used for tree inference with IQ-TREE 2. Optimal substitution models: TVM+F+R6 (**A**) and (**B**), and TVMe+R7 (**C**). The trees in panels A and B were arbitrarily rooted with members of the family *Matonaviridae*, and in panel (**C**), with members of the family *Astroviridae* and astro-like viruses. Scale bars indicate substitutions per site. Teltowkanal hepe-like viruses identified in consecutive samples are indicated by a diamond (◆). Triangles (▲) and dots (●) indicate viruses from this study with almost complete and partial genome sequences, respectively. Details of the trees are presented in Supplementary Figure S2

The tree topologies in the three phylogenetic analyses demonstrate several robust clades in addition to the families *Alphatetraviridae*, *Benyviridae*, *Hepeviridae*, *Matonaviridae*, and *Astroviridae*. The first clade includes human and animal bastroviruses, which always clustered in two subclades. The second clade, the so-called ‘bastro-like’ viruses, which include HHLV-6, -10, -15, and -16 and TkHLV-28, -29, -30, -31, and -38 from our water samples, were always located apart from the bastroviruses but close to the alphatetraviruses (Fig. 1). The genomes of bastro-like viruses contain two ORFs. In addition to the phylogenetic results and the fact that they have a different host, which is likely a non-vertebrate, 'bastro-like' viruses are distinguished from the human and animal bastroviruses by the presence of a non-homologous CP. This is of particular interest, as the CP of human and animal bastroviruses exhibits a striking similarity to the astrovirus CP, whereas the CP of ‘bastro-like’ viruses does not (Supplementary Fig. 3). A third clade is comprised of up to 11 viruses of arthropods, HHLV-8, and TkHLV-22, -32, and -33. These viruses share one characteristic feature: an RdRp with permuted palm motifs. Whereas all positive-stranded RNA viruses with a canonical RdRp have seven conserved structural motifs (consisting of β-strands and α-helices) in the order G-F-A-B-C-D-E [11], the 11 hepe-like viruses of this clade exhibit the permuted order -C-A-B- of their active site motifs (Fig. 2 and Supplementary Fig. S5). The genomes of Beihai barnacle virus 1, Changjiang hepe-like virus 1, and Beijing sediment hepe-like virus 1 encode four hypothetical proteins in addition to the polyprotein and have sizes of 10-11 kb, which is considerable larger than the genomes of the monopartite viruses of the acknowledged *Hepelivirales* families. This genome layout, however, is not conserved. TkHLV-32 and -33 have only three ORFs, with ORF1 and 2 corresponding to ORFs 1 and 4 of the former viruses.

**Fig. 2:**
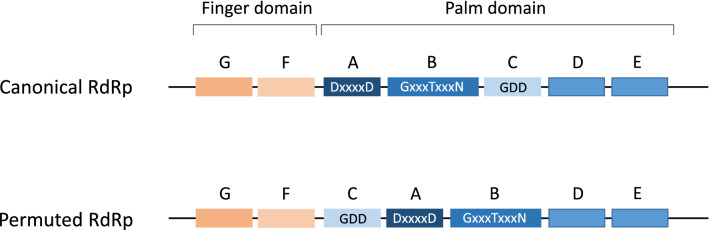
Schematic presentation of RNA-dependent RNA polymerases (RdRp) with the canonical and permuted order of the conserved palm motifs. Blue boxes represent motifs A to E of the palm subdomain, and brown boxes represent motifs G and F of the finger subdomain. DxxxxD, GxxxTxxxN, and GDD indicate conserved amino acids of the RdRp active site

Nine hepeliviruses were found to have peptidase-like CPs (Supplementary Fig. S4), and six possessed a third ORF encoding a protein with a Zn-binding RING/Ubox domain (data not shown).

We expected to trace human and animal enteric viruses, as all of the samples were collected in the metropolitan area of Berlin, Germany, and the two waterbodies are linked and receive the discharge of a local wastewater treatment plant and the drain water effluents of the city of Berlin after periods of heavy rainfall. Although such viruses are detectable in Havel River and Teltow Canal samples by specific PCR methods throughout the year [2 and unpublished results], we failed to identify sequence reads of known pathogenic noroviruses (*Caliciviridae*), enteroviruses (*Picornaviridae*), astroviruses (*Astroviridae*), or hepatitis A or hepatitis E viruses (*Picornaviridae*, *Hepeviridae*) in our metagenomes. Instead, we were able to demonstrate the presence of many unclassified hepe-like viruses and a few astroviruses. As shown recently for the picorna-like viruses of the Havel River [21], this result indicates the presence of a vast number of uncultured viruses compared to the still moderate number of viruses in the known virosphere. One notable group of unclassified hepe-like viruses are the bastroviruses, which can be differentiated in phylogenetic analysis into human bastroviruses with three ORFs and animal bastroviruses with two ORFs (Fig. 1). These viruses likely have vertebrate hosts. Another characteristic feature of bastroviruses is a CP with striking similarity to the astrovirus CP (Supplementary Fig. S3). The significance of this similarity, as well as the prevalence of bastroviruses in vertebrates remains to be elucidated. One member of this group, the Guangdong fish caecilians hepevirus, however, exhibits more variation in the CP gene than in the domains of the nonstructural protein (Supplementary Figs. S2 and S3). Overall, the four *Hepelivirales* families, the bastroviruses, and the 'bastro-like' viruses each have unique structural proteins with little or no similarity to one other.

Another clade of hepe-like viruses (Fig. 1) is comprised of the so-called ‘bastro-like’ viruses. They have rather small genomes (<7 kb) that are significantly different from those of the bastroviruses. Some bastro-like viruses have been detected in insectivore bats and insects (*Culex* mosquitoes, wasps), while others have been found in aquatic organisms (sponges, bivalves, fish). The presence of bastro-like viruses in environmental water samples from the Havel River and Teltow Canal is compatible with the assumption that these viruses have aquatic hosts.

A third interesting clade includes viruses with rather large genomes (up to 11 kb). These viruses have been detected in mantis flies, crayfish, and barnacles and in various environmental samples, including our Havel River and Teltow Canal water samples. As shown in Fig. 2, the RdRp of these viruses has a permuted order of the conserved palm subdomain motifs. A permuted RdRp has been observed only in birnaviruses [11] and two permutotetraviruses (Thosea asigna virus and Euprosterna elaeasa virus) [6]. In addition to the permuted order of the RdRp palm subdomain motifs, the polymerase sequences of the members of the families *Birnaviridae* and *Permutotetraviridae* differ significantly from those of the alphavirus supergroup, which includes the hepeliviruses. To our knowledge, this study is the first description of members of the alphavirus supergroup with a permuted RdRp.

One obstacle to metagenomic studies of environmental water samples is the lack of information on the virus hosts. Even virus detection in faecal samples or the gills and guts of fish, arthropods, or molluscs may indicate dietary uptake or accumulation by filtration rather than infection. However, the increasing number of hepeliviruses associated with non-vertebrates suggests that they are the actual hosts. Further, the similarity of sequences from various sources and locations suggests a similar host range and a global distribution of hepeliviruses in terrestrial, marine, and freshwater ecosystems, which clearly deserves a more extensive investigation regarding prevalence and host range. Recurrent detection of identical viruses in samples from two linked waterbodies sampled in consecutive years, as shown in the present study, suggests an enzootic prevalence of hepeliviruses in many unknown hosts.

## Supplementary Information

Below is the link to the electronic supplementary material.

Supplementary file1 Supplementary Fig. S1 Genome organisation of Havel hepe-like viruses (HHLV) and Teltowkanal hepe-like viruses (TkHLV). Conserved domains and sequence motifs were identified by searching the NCBI Conserved Domain Database (CDD, https://www.ncbi.nlm.nih.gov/Structure/cdd/wrpsb.cgi). Green boxes represent open reading frames (ORFs) without detectable conserved domains. Abbreviations: UTR, untranslated region; VMethTr, viral methyltransferase; Hel, helicase; RdRp, polymerase; FtsJ, FtsJ-like methyltransferase; Macro, macrodomain with similarity to the X-domain; PeptA6, putative capsid protein with similarity to members of the peptidase A6 family; rhv, putative capsid protein with similarity to the rhinovirus capsid protein, with a jelly-roll fold and a drug-binding pocket; Ubox, U-box protein with modified RING finger domain that lacks some zinc-binding residues. Supplementary Fig. S2 Phylogenetic analysis of 105 viral N7-methyltransferase sequences (A), 107 helicase sequences (B), and 130 RdRp sequences (C) of hepeliviruses. Sequences of Havel hepe-like viruses (HHLV; printed in red), Teltowkanal hepe-like viruses (TkHLV; printed in green), unclassified hepe-like viruses (printed in black), and reference strains of the families Alphatetraviridae, Benyviridae, Hepeviridae, Matonaviridae, and Astroviridae (printed in blue) were aligned with MEGA and used for tree inference with IQ-TREE 2. Optimal substitution models: TVM+F+R6 (A) and (B) and TVMe+R7 (C). The trees in panels A and B were arbitrarily rooted with members of the family Matonaviridae, and in panel C with members of the family Astroviridae and astro-like viruses. Blue boxes indicate reference virus families; yellow, brown, and green boxes denote unclassified bastroviruses, 'bastro-like' viruses, and a clade of hepe-like viruses with permuted RdRp. Scale bars indicate substitutions per site. Teltowkanal hepe-like viruses, which were detected in consecutive samples from 2016, 2017 and 2018, are indicated by a diamond (◆). Triangles (▲) and dots (●) indicate viruses from this study with almost complete and partial genome sequences, respectively. Given are GenBank accession numbers, genus names (printed in italics) of classified reference viruses, virus names, and strain designations (in brackets) where available. Numbers at nodes indicate bootstrap values obtained after 50,000 ultrafast bootstrap replications. The bar indicates substitutions per site. Supplementary Fig. S3 Phylogenetic analysis of capsid protein sequences. The blue box indicates reference viruses; yellow and brown boxes denote unclassified bastroviruses and 'bastro-like' viruses. Sequences of Havel hepe-like viruses (HHLV; printed in red), bastroviruses, bastro-like and astro-like viruses (all printed in black), and reference strains of the family Astroviridae (printed in blue) were aligned in MEGA and used for tree inference with IQ-TREE 2. Optimal substitution model: TVM+F+R4. The scale bar indicates substitutions per site. The triangle (▲) and dots (●) indicate HHLVs with complete and partial genome sequences, respectively. Note that Guangdong fish caecilians hepevirus, an animal bastrovirus, clusters with astroviruses. Supplementary Fig. S4 Phylogenetic analysis of viral capsid proteins with similarity to peptidases A6 and A21. Capsid protein sequences of representative members of the families Alphatetraviridae and Permutotetraviridae (printed in blue) and unclassified viruses (printed in black) including Havel hepe-like viruses and Teltowkanal hepe-like viruses (printed in red) were used for tree inference with IQTree (optimal substitution model: TVM+F+G4). Prior to phylogenetic analysis, similarity to peptidases A6 or A21 was verified by a CDD search. Numbers at nodes indicate bootstrap support obtained after 50,000 ultrafast bootstrap replications. The bar indicates substitutions per site. The green and ochre boxes indicate viruses with capsid proteins with similarity to peptidase A6 and A21, respectively. Note the unexpected clustering of Teltowkanal hepe-like virus 24 (TkHLV-24), which has a capsid protein with similarity to peptidase A6. Supplementary Fig. S5 Partial alignment of 50 hepelivirus RdRp sequences. Representative sequences of the four hepelivirus families (printed in blue), unclassified hepeliviruses (printed in black), and a clade of 11 hepeliviruses with permuted RdRp palm motifs (printed in red) were aligned using MEGA. Hyphens indicate gaps or missing data. Three conserved palm domain motifs of the RdRp, named A, B and C, are highlighted in green. Highly conserved amino acids of the RdRp active site are highlighted in yellow, i.e., DxxxxD in motif A, SGxxxTxxxN in motif B, and GDD in motif C. (PDF 2215 KB)

## Data Availability

The BioProject was submitted to the BioProject Database: BioProject ID PRJNA889576. Sequence reads were submitted to the Short Read Archive of NCBI: SRR2189516, SRR2189517, SRR2189518, SRR2189519.BioSamples were submitted to BioSample Database: acc. nos. SAMN31248992, SAMN31248993, SAMN31248994, SAMN31248995. Release date: Sept. 12th, 2022.Sequence data were submitted to GenBank: acc. nos. OP699055 - OP699153. We received a statement by GenBank staff that sequences will be released on Dec. 28th, 2022.
