# Supplementary material for: Hepeliviruses in two waterbodies in Berlin, Germany
Source: Arch Virol. 2022 Dec 25;168(1):9. doi: 10.1007/s00705-022-05688-0 (PMC9790848; doi:10.1007/s00705-022-05688-0)
Supplement: Supplementary file 1 — Supplementary file1 Supplementary Fig. S1 Genome organisation of Havel hepe-like viruses (HHLV) and Teltowkanal hepe-like viruses (TkHLV). Conserved domains and sequence motifs were identified by searching the NCBI Conserved Domain Database (CDD, https://www.ncbi.nlm.nih.gov/Structure/cdd/wrpsb.cgi). Green boxes represent open reading frames (ORFs) without detectable conserved domains. Abbreviations: UTR, untranslated region; VMethTr, viral methyltransferase; Hel, helicase; RdRp, polymerase; FtsJ, FtsJ-like methyltransferase; Macro, macrodomain with similarity to the X-domain; PeptA6, putative capsid protein with similarity to members of the peptidase A6 family; rhv, putative capsid protein with similarity to the rhinovirus capsid protein, with a jelly-roll fold and a drug-binding pocket; Ubox, U-box protein with modified RING finger domain that lacks some zinc-binding residues. Supplementary Fig. S2 Phylogenetic analysis of 105 viral N7-methyltransferase sequences (A), 107 helicase sequences (B), and 130 RdRp sequences (C) of hepeliviruses. Sequences of Havel hepe-like viruses (HHLV; printed in red), Teltowkanal hepe-like viruses (TkHLV; printed in green), unclassified hepe-like viruses (printed in black), and reference strains of the families Alphatetraviridae, Benyviridae, Hepeviridae, Matonaviridae, and Astroviridae (printed in blue) were aligned with MEGA and used for tree inference with IQ-TREE 2. Optimal substitution models: TVM+F+R6 (A) and (B) and TVMe+R7 (C). The trees in panels A and B were arbitrarily rooted with members of the family Matonaviridae, and in panel C with members of the family Astroviridae and astro-like viruses. Blue boxes indicate reference virus families; yellow, brown, and green boxes denote unclassified bastroviruses, 'bastro-like' viruses, and a clade of hepe-like viruses with permuted RdRp. Scale bars indicate substitutions per site. Teltowkanal hepe-like viruses, which were detected in consecutive samples from 2016, 2017 and 2018, a [file 705_2022_5688_MOESM1_ESM.pdf]

Hepeliviruses of two waterbodies in Berlin, Germany

Roland Zell, Marco Groth, Lukas Selinka, Hans-Christoph Selinka

Supplementary Material

Legends to Supplementary Figures

Supplementary Figure 1: Genome organisation of Havel hepe-like viruses (HHLV) and Teltowkanal hepe-like viruses (TkHLV).
Conserved domains and sequence motifs were identified by searching the NCBI Conserved Domain Database (CDD, https://www.ncbi.nlm.nih.gov/Structure/cdd/wrpsb.cgi). Green boxes represent open reading frames (ORFs) without detectable conserved domains. **Abbreviations:** UTR, untranslated region; VMethTr, viral methyltransferase; Hel, helicase; RdRP, polymerase; FtsJ, FtsJ-like methyltransferase; Macro, macrodomain with similarity to the X-domain; PeptA6, putative capsid protein with similarity to peptidase A6 family; rhv, putative capsid protein with similarity to the rhinovirus capsid protein with jelly roll fold and drug-binding pocket; Ubox, U-box protein with modified RING finger domain that lacks some zink binding residues.

Supplementary Figure 2: Phylogenetic analysis of 105 viral N7-methyltransferase sequences (A), 107 helicase sequences (B), and 130 RdRP sequences (C) of hepeliviruses.
Sequences of Havel hepe-like viruses (HHLV; printed in red), Teltowkanal hepe-like viruses (TkHLV; printed in green), unclassified hepe-like viruses (printed in black) and reference strains of the families *Alphatetraviridae*, *Benyviridae*, *Hepeviridae*, *Matonaviridae*, and *Astroviridae* (printed in blue) were aligned with Mega and used for tree inference with IQ-TREE 2. Optimal substitution models: TVM+F+R6 (A) and (B), and TVMe+R7 (C). The trees in (A) and (B) were arbitrarily rooted with the *Matonaviridae*, in (C) with *Astroviridae* and astro-like viruses. Blue boxes indicate reference virus families; yellow, brown and green boxes denote unclassified bastroviruses, 'bastro-like' viruses and a clade of hepe-like viruses with permuted RdRP. Scale bars indicate substitutions per site. Teltowkanal hepe-like viruses which were detected in consecutive samples of 2016, 2017 and 2018 are labelled with a diamond (♦). Triangles (▲) and dots (●) indicate viruses of this study with almost complete and partial genomes, respectively. Given are GenBank accession numbers, genus names (printed in italics) of classified reference viruses, virus names and strain designations (in brackets) where available. Numbers at nodes indicate bootstrap values obtained after 50,000 ultrafast bootstrap replications. The bar indicates the substitutions per site.

Supplementary Figure 3: Phylogenetic analysis of capsid protein sequences of astroviruses, bastroviruses and 'bastro-like' viruses.
The blue box indicates reference viruses; yellow and brown boxes denote unclassified bastroviruses and 'bastro-like' viruses. Sequences of Havel hepe-like viruses (HHLV; printed in red), bastroviruses, bastro-like and astro-like viruses (all printed in black) and reference strains of the family *Astroviridae* (printed in blue) were aligned with Mega and used for tree inference with IQ-TREE 2. Optimal substitution model: TVM+F+R4. The scale bar indicates substitutions per site. The triangle (▲) and dots (●) indicate HHLVs with complete and partial genomes, respectively. Note that Guangdong fish caecilians hepevirus, an animal bastrovirus, clusters with astroviruses.

Supplementary Figure 4: Phylogenetic analysis of viral capsid proteins with similarity to peptidases A6 and A21.
Capsid protein sequences of representative members of the families *Alphatetraviridae* and *Permutotetraviridae* (printed in blue) and unclassified viruses (printed in black) including Havel hepe-like viruses and Teltowkanal hepe-like viruses (printed in red) were used for tree inference with IQTree (optimal substitution model: TVM+F+G4). Prior to phylogenetic analysis, similarity to peptidases A6 or A21 was verified by a CDD search. Numbers at nodes indicate bootstrap support obtained after 50,000 ultrafast bootstrap replications. The bar indicates substitutions per site. The green and ochre boxes indicate viruses with capsid proteins with similarity to peptidase A6 and A21, respectively. Note the

unexpected clustering of Teltowkanal hepe-like virus 14 (TkHLV-14) which exhibits a capsid protein with similarity to peptidase A6.

Supplementary Figure 5: Partial alignment of 50 hepelivirus RdRP sequences.
Representative sequences of the four hepelivirus families (printed in blue), unclassified hepeliviruses (printed in black) and a clade of 11 hepeliviruses with permuted RdRP palm motifs (printed in red) were aligned with Mega. Hyphens indicate gaps or missing data. Three conserved palm domain motifs of the RdRP, named A, B and C, are highlighted in green. Highly conserved amino acids of the RdRP active site are highlighted in yellow, i.e., DxxxxD in motif A, SGxxxTxxxN in motif B and GDD in motif C.

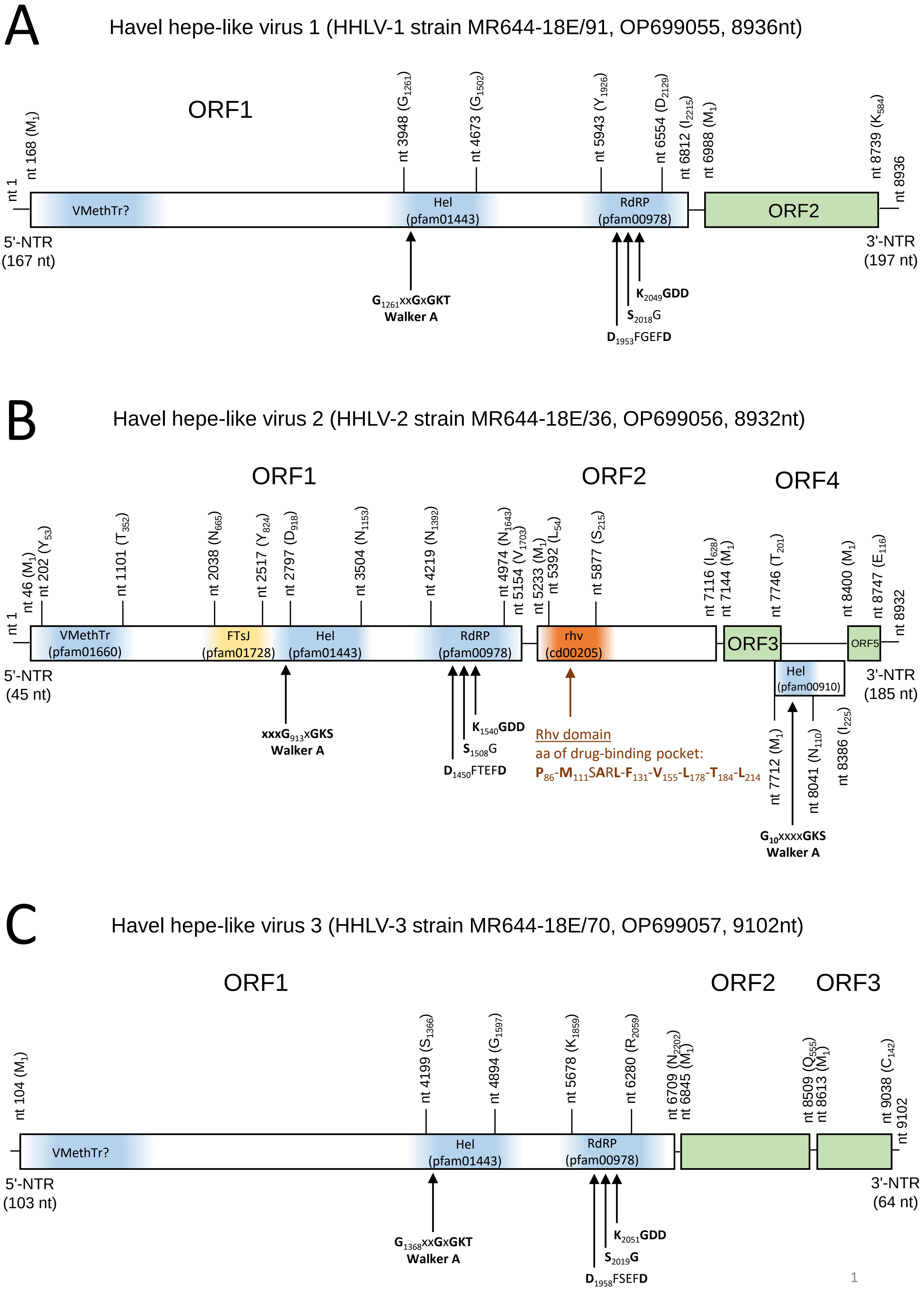
A
Havel hepe-like virus 1 (HHLV-1 strain MR644-18E/91, OP699055, 8936nt)
ORF1
nt 1
nt 168 (M_1)
nt 3948 (G_{1261})
nt 4673 (G_{1502})
nt 5943 (Y_{1926})
nt 6554 (D_{2129})
nt 6812 (I_{2215})
nt 6988 (M_1)
nt 8739 (K_{584})
nt 8936
VMethTr?
Hel
(pfam01443)
RdRP
(pfam00978)
ORF2
5'-NTR
(167 nt)
3'-NTR
(197 nt)
G_{1261}xxGxGKT
Walker A
K_{2049}GDD
S_{2018}G
D_{1953}FGEFD
B
Havel hepe-like virus 2 (HHLV-2 strain MR644-18E/36, OP699056, 8932nt)
ORF1
ORF2
ORF4
nt 1
nt 46 (M_1)
nt 202 (Y_{53})
nt 1101 (T_{352})
nt 2038 (N_{665})
nt 2517 (Y_{824})
nt 2797 (D_{918})
nt 3504 (N_{1153})
nt 4219 (N_{1392})
nt 4974 (N_{1643})
nt 5154 (V_{1703})
nt 5233 (M_1)
nt 5392 (L_{54})
nt 5877 (S_{215})
nt 7116 (I_{628})
nt 7144 (M_1)
nt 7746 (T_{201})
nt 8400 (M_1)
nt 8747 (E_{116})
nt 8932
VMethTr
(pfam01660)
FTsJ
(pfam01728)
Hel
(pfam01443)
RdRP
(pfam00978)
rhv
(cd00205)
ORF3
ORF5
Hel
(pfam00910)
5'-NTR
(45 nt)
3'-NTR
(185 nt)
xxxG_{913}xGKS
Walker A
K_{1540}GDD
S_{1508}G
D_{1450}FTEFD
Rhv domain
aa of drug-binding pocket:
P_{86}-M_{111}SARL-F_{131}-V_{155}-L_{178}-T_{184}-L_{214}
nt 7712 (M_1)
nt 8041 (N_{110})
nt 8386 (I_{225})
G_{10}xxxxGKS
Walker A
C
Havel hepe-like virus 3 (HHLV-3 strain MR644-18E/70, OP699057, 9102nt)
ORF1
ORF2
ORF3
nt 104 (M_1)
nt 4199 (S_{1366})
nt 4894 (G_{1597})
nt 5678 (K_{1859})
nt 6280 (R_{2059})
nt 6709 (N_{2202})
nt 6845 (M_1)
nt 8509 (Q_{555})
nt 8613 (M_1)
nt 9038 (C_{142})
nt 9102
VMethTr?
Hel
(pfam01443)
RdRP
(pfam00978)
5'-NTR
(103 nt)
3'-NTR
(64 nt)
G_{1368}xxGxGKT
Walker A
K_{2051}GDD
S_{2019}G
D_{1958}FSEFD

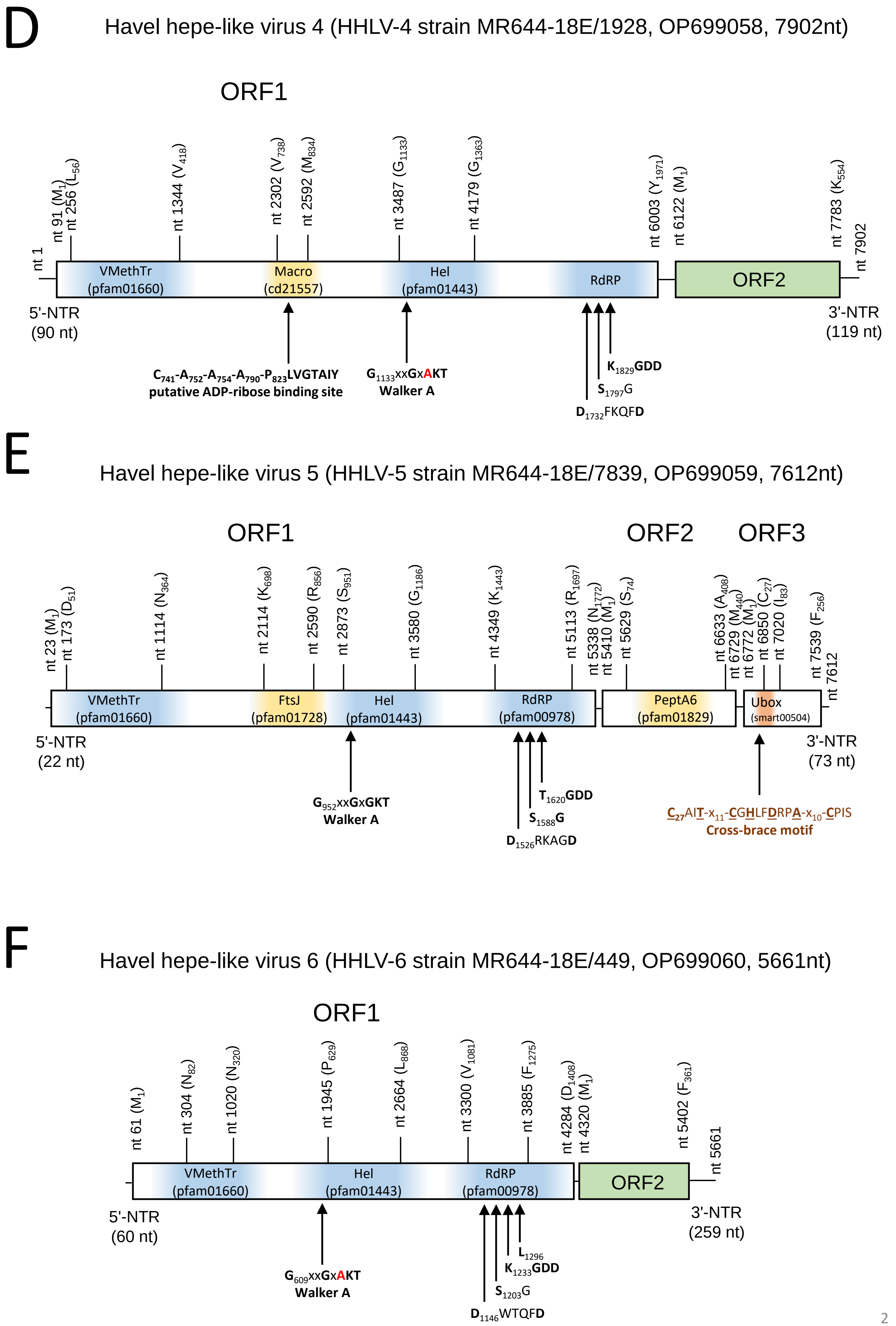
D
Havel hepe-like virus 4 (HHLV-4 strain MR644-18E/1928, OP699058, 7902nt)
ORF1
nt 1
nt 91 (M1)
nt 256 (L56)
nt 1344 (V418)
nt 2302 (V738)
nt 2592 (M834)
nt 3487 (G1133)
nt 4179 (G1363)
nt 6003 (Y1971)
nt 6122 (M1)
nt 7783 (K554)
nt 7902
VMethTr (pfam01660)
Macro (cd21557)
Hel (pfam01443)
RdRP
ORF2
5'-NTR (90 nt)
3'-NTR (119 nt)
C741-A752-A754-A790-P823LVGTAIY putative ADP-ribose binding site
G1133xxGxAKT Walker A
K1829GDD
S1797G
D1732FKQFD
E
Havel hepe-like virus 5 (HHLV-5 strain MR644-18E/7839, OP699059, 7612nt)
ORF1
ORF2
ORF3
nt 23 (M1)
nt 173 (D51)
nt 1114 (N364)
nt 2114 (K698)
nt 2590 (R856)
nt 2873 (S951)
nt 3580 (G1186)
nt 4349 (K1443)
nt 5113 (R1697)
nt 5338 (N1772)
nt 5410 (M1)
nt 5629 (S74)
nt 6633 (A408)
nt 6729 (M440)
nt 6772 (M1)
nt 6850 (C27)
nt 7020 (I83)
nt 7539 (F256)
nt 7612
VMethTr (pfam01660)
FtsJ (pfam01728)
Hel (pfam01443)
RdRP (pfam00978)
PeptA6 (pfam01829)
Ubox (smart00504)
5'-NTR (22 nt)
3'-NTR (73 nt)
G952xxGxGKT Walker A
T1620GDD
S1588G
D1526RKAGD
C27AIT-x11-CGHLFDRPA-x10-CPIS Cross-brace motif
F
Havel hepe-like virus 6 (HHLV-6 strain MR644-18E/449, OP699060, 5661nt)
ORF1
nt 61 (M1)
nt 304 (N82)
nt 1020 (N320)
nt 1945 (P629)
nt 2664 (L868)
nt 3300 (V1081)
nt 3885 (F1275)
nt 4284 (D1408)
nt 4320 (M1)
nt 5402 (F361)
nt 5661
VMethTr (pfam01660)
Hel (pfam01443)
RdRP (pfam00978)
ORF2
5'-NTR (60 nt)
3'-NTR (259 nt)
G609xxGxAKT Walker A
L1296
K1233GDD
S1203G
D1146WTQFD

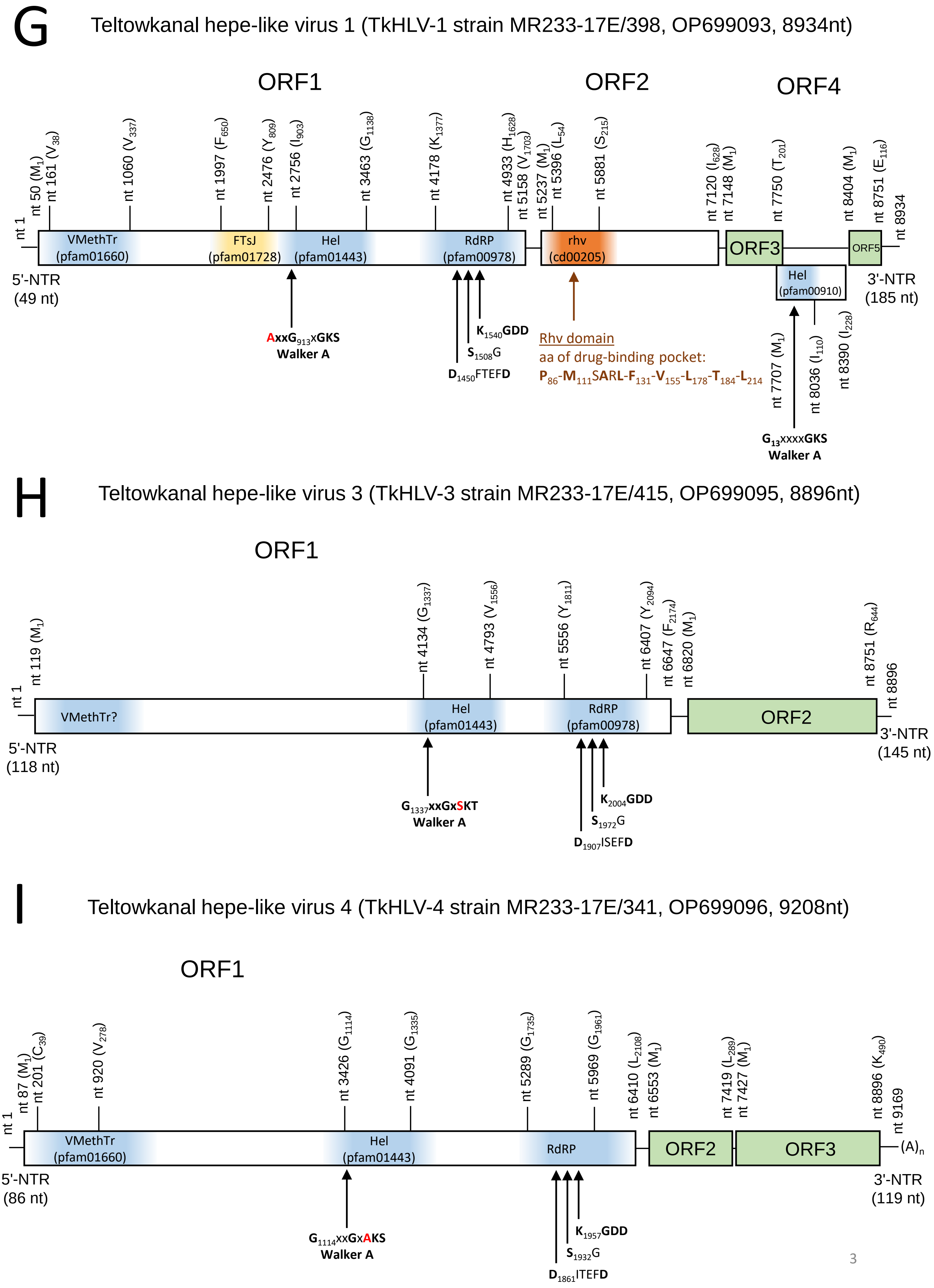
G
Teltowkanal hepe-like virus 1 (TkHLV-1 strain MR233-17E/398, OP699093, 8934nt)
ORF1
ORF2
ORF4
nt 1
nt 50 (M_1)
nt 161 (V_{38})
nt 1060 (V_{337})
nt 1997 (F_{650})
nt 2476 (Y_{809})
nt 2756 (I_{903})
nt 3463 (G_{1138})
nt 4178 (K_{1377})
nt 4933 (H_{1628})
nt 5158 (V_{1703})
nt 5237 (M_1)
nt 5396 (L_{54})
nt 5881 (S_{215})
nt 7120 (I_{628})
nt 7148 (M_1)
nt 7750 (T_{201})
nt 8404 (M_1)
nt 8751 (E_{116})
nt 8934
VMethTr (pfam01660)
FTsJ (pfam01728)
Hel (pfam01443)
RdRP (pfam00978)
rhv (cd00205)
ORF3
ORF5
Hel (pfam00910)
5'-NTR (49 nt)
3'-NTR (185 nt)
AxxG$_{913}$xGKS
Walker A
K_{1540}GDD
S_{1508}G
D_{1450}FTEFD
Rhv domain
aa of drug-binding pocket:
P_{86}-M_{111}SARL-F_{131}-V_{155}-L_{178}-T_{184}-L_{214}
nt 7707 (M_1)
nt 8036 (I_{110})
nt 8390 (I_{228})
G_{13}xxxxGKS
Walker A
H
Teltowkanal hepe-like virus 3 (TkHLV-3 strain MR233-17E/415, OP699095, 8896nt)
ORF1
nt 1
nt 119 (M_1)
nt 4134 (G_{1337})
nt 4793 (V_{1556})
nt 5556 (Y_{1811})
nt 6407 (Y_{2094})
nt 6647 (F_{2174})
nt 6820 (M_1)
nt 8751 (R_{644})
nt 8896
VMethTr?
Hel (pfam01443)
RdRP (pfam00978)
ORF2
5'-NTR (118 nt)
3'-NTR (145 nt)
G_{1337}xxGxSKT
Walker A
K_{2004}GDD
S_{1972}G
D_{1907}ISEFD
I
Teltowkanal hepe-like virus 4 (TkHLV-4 strain MR233-17E/341, OP699096, 9208nt)
ORF1
nt 1
nt 87 (M_1)
nt 201 (C_{39})
nt 920 (V_{278})
nt 3426 (G_{1114})
nt 4091 (G_{1335})
nt 5289 (G_{1735})
nt 5969 (G_{1961})
nt 6410 (L_{2108})
nt 6553 (M_1)
nt 7419 (L_{289})
nt 7427 (M_1)
nt 8896 (K_{490})
nt 9169
VMethTr (pfam01660)
Hel (pfam01443)
RdRP
ORF2
ORF3
$(A)_n$
5'-NTR (86 nt)
3'-NTR (119 nt)
G_{1114}xxGxAKS
Walker A
K_{1957}GDD
S_{1932}G
D_{1861}ITEFD

J

Teltowkanal hepe-like virus 8 (TkHLV-8 strain MR233-17E/430, OP699101, 8817nt)

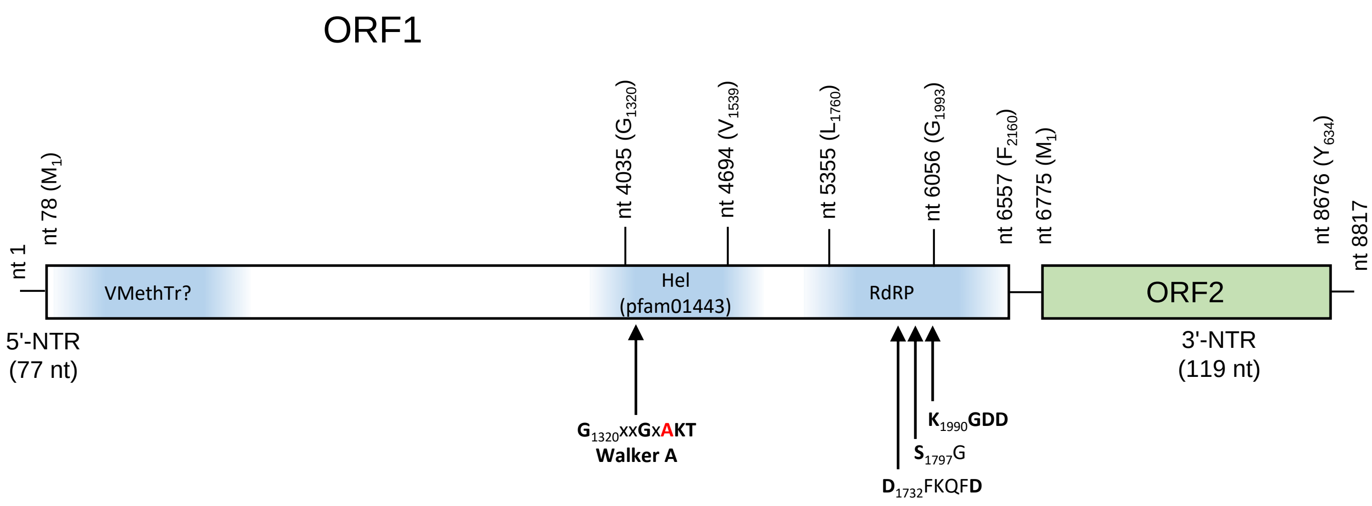

K

Teltowkanal hepe-like virus 12(TkHLV-12 strain MR233-17E/582, OP699107, 8061nt)

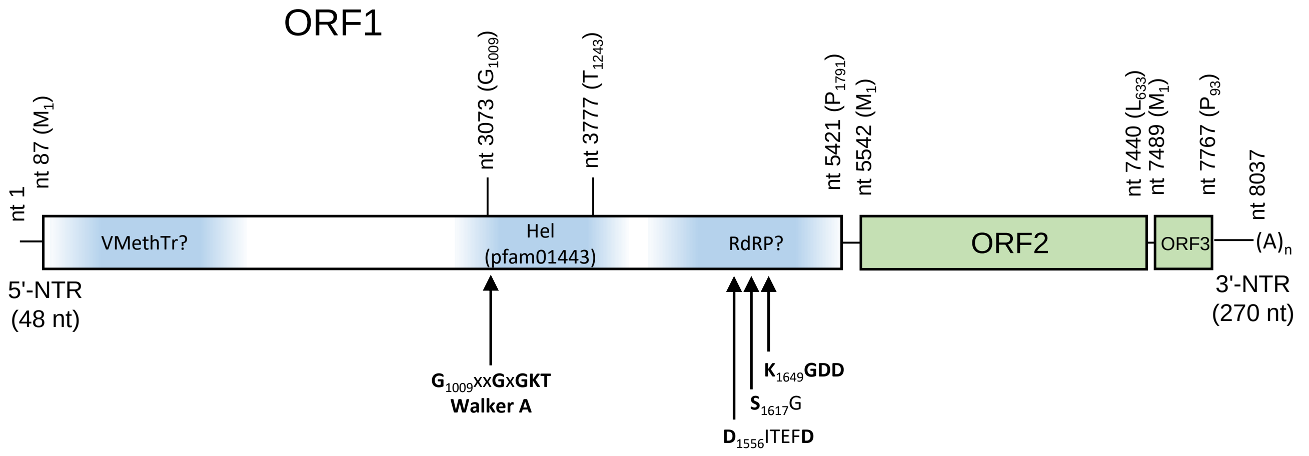

L

Teltowkanal hepe-like virus 16 (TkHLV-16 strain MR233-17E/325, OP699112, 9268nt)

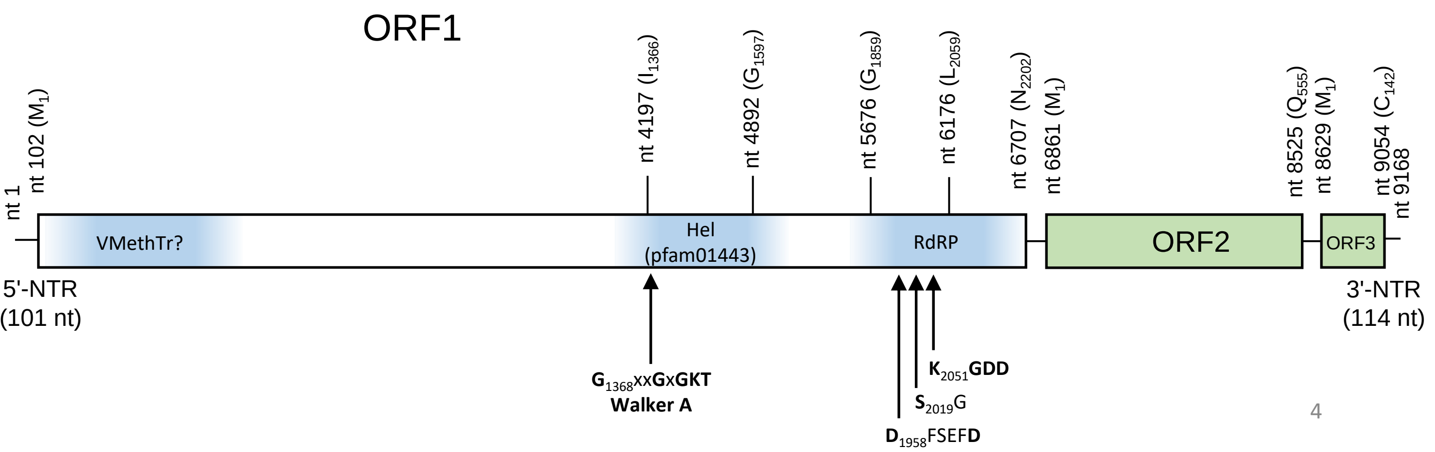

M Teltowkanal hepe-like virus 18 (TkHLV-18 strain 233-17E/349, OP699115, 9184nt)

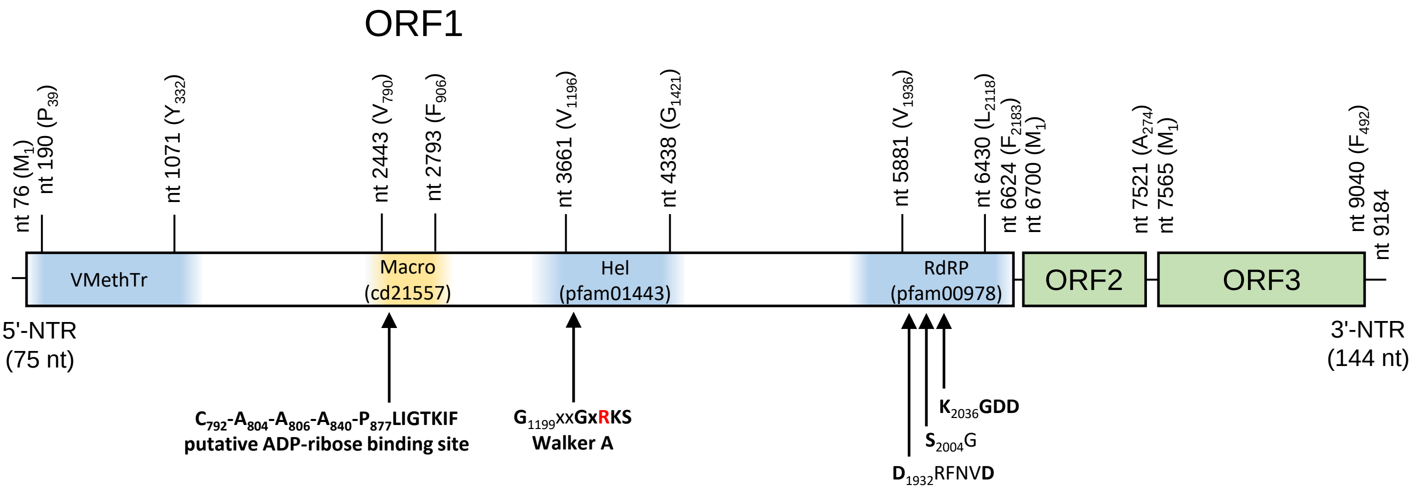

N Teltowkanal hepe-like virus 20 (TkHLV-20 strain MR233-17E/643, OP699118, 7758nt)

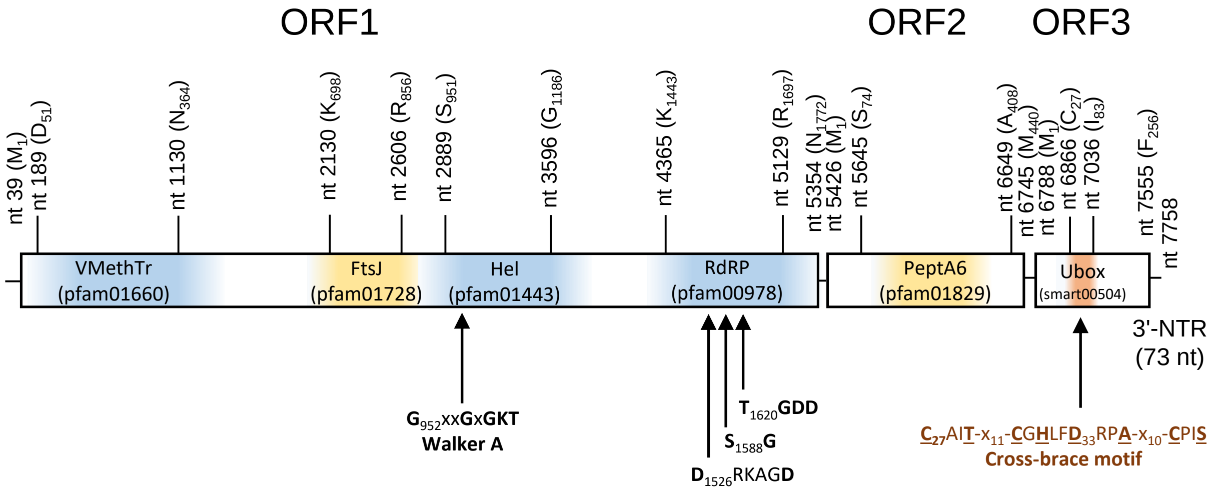

O Teltowkanal hepe-like virus 22 (TkHLV-22 strain MR233-17E/663, OP699120, 7653nt)

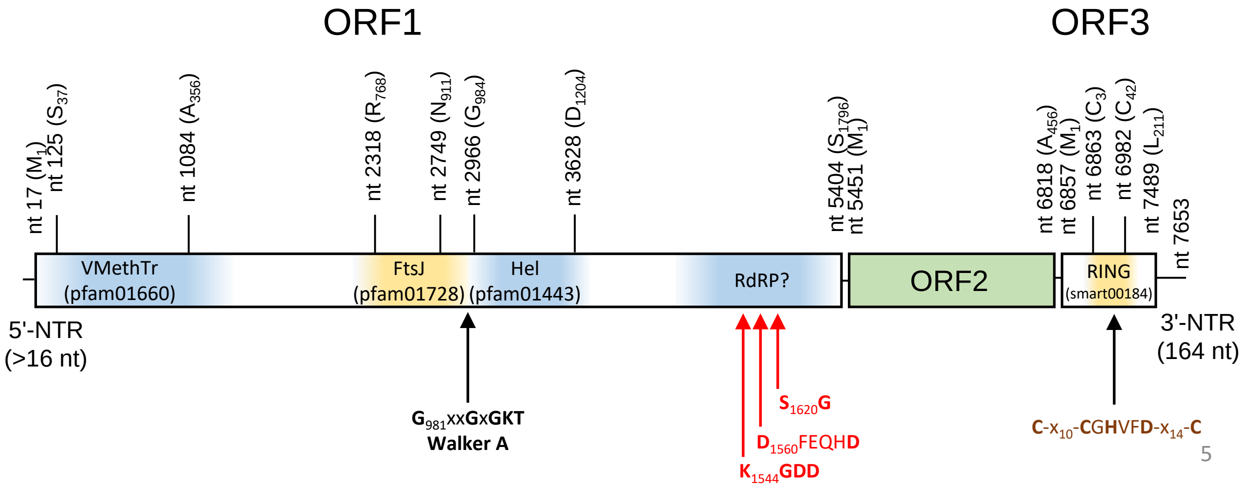

P Teltowkanal hepe-like virus 25 (TkHLV-25 strain 233-17E/401, OP699123, 8927nt)

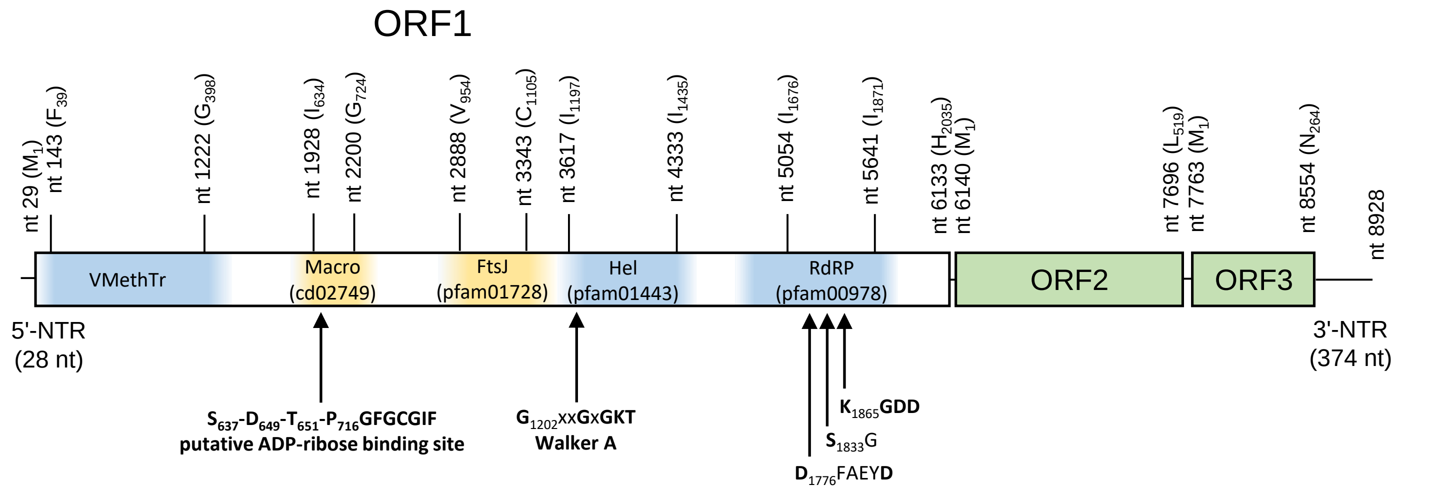

Q Teltowkanal hepe-like virus 32 (TkHLV-32 strain MR233-17E/3264, OP699130, 9266nt)

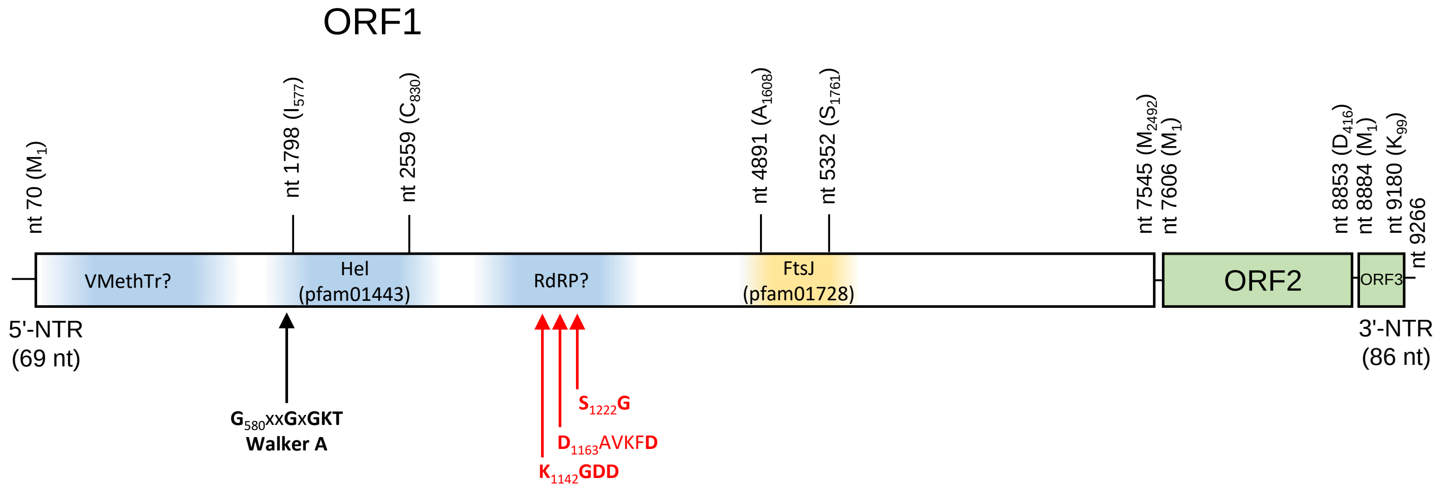

R Teltowkanal hepe-like virus 33 (TkHLV-33 strain MR233-17E/476, OP699131, 8587nt)

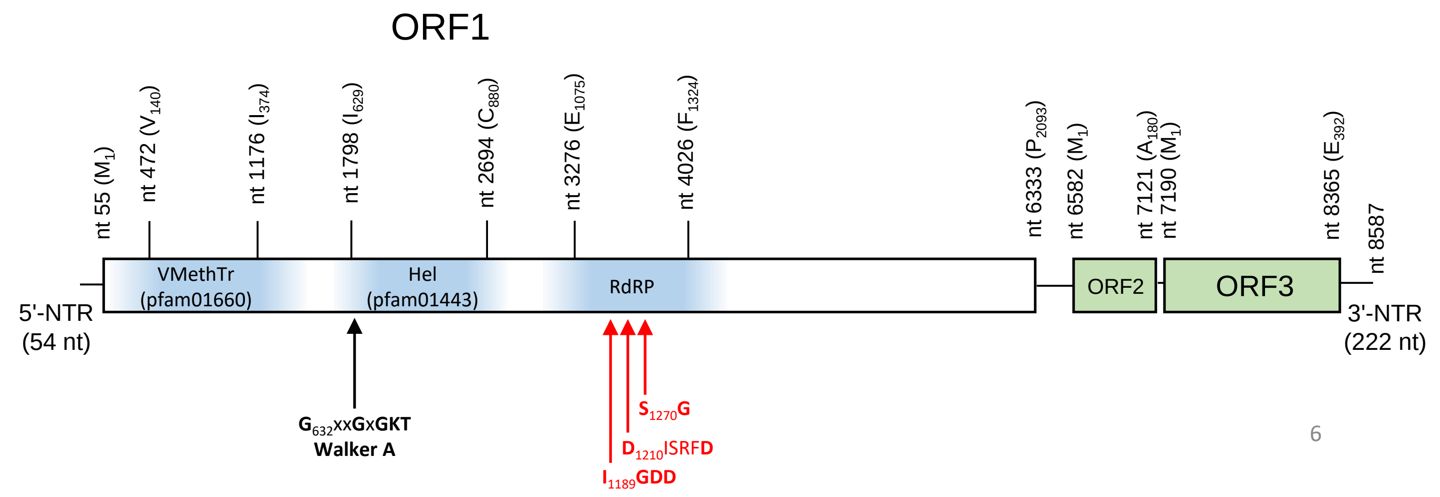

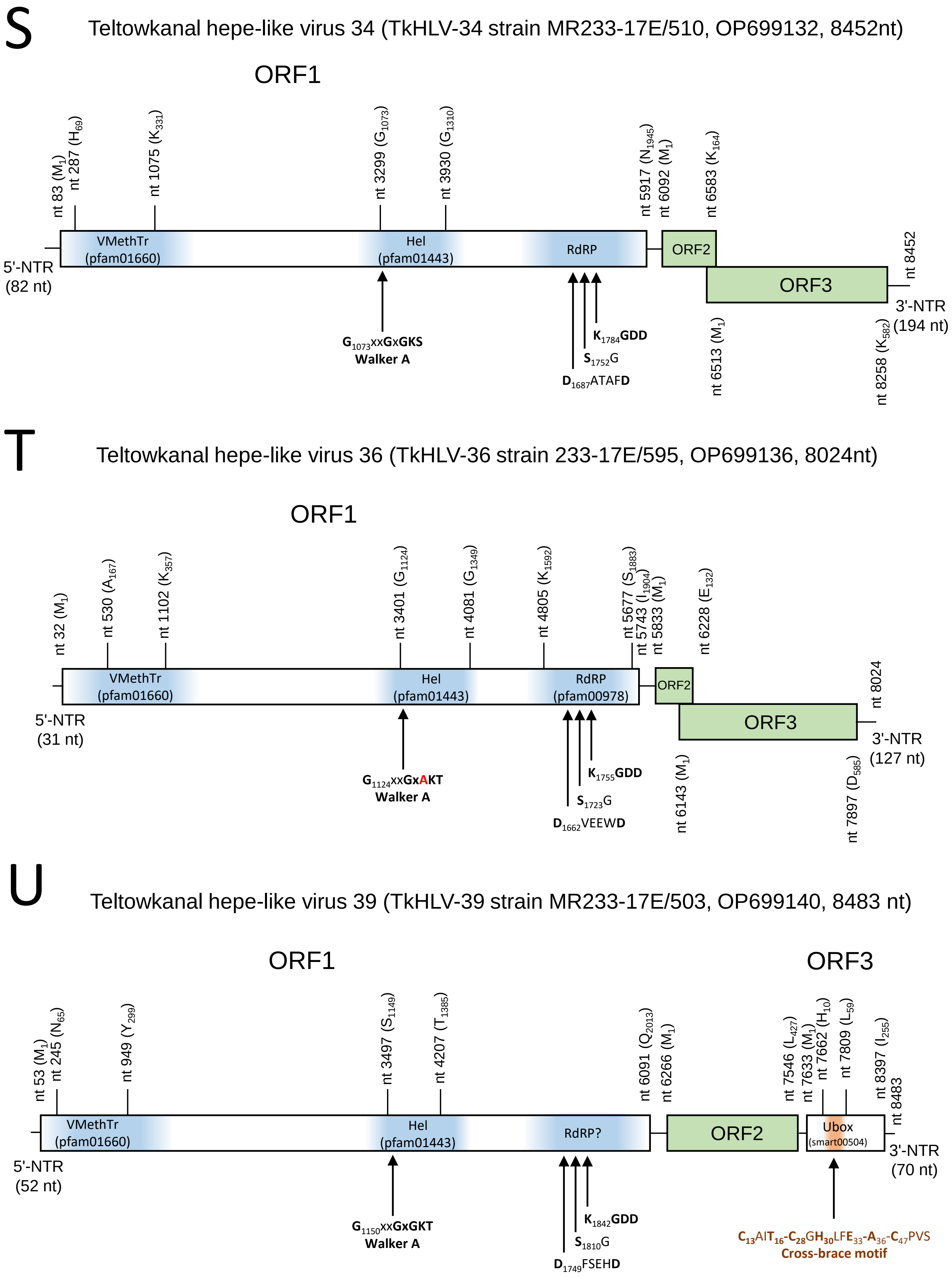
S
Teltowkanal hepe-like virus 34 (TkHLV-34 strain MR233-17E/510, OP699132, 8452nt)
ORF1
nt 83 (M1)
nt 287 (H69)
nt 1075 (K331)
nt 3299 (G1073)
nt 3930 (G1310)
nt 5917 (N1945)
nt 6092 (M1)
nt 6583 (K164)
VMethTr (pfam01660)
Hel (pfam01443)
RdRP
ORF2
ORF3
nt 8452
5'-NTR (82 nt)
3'-NTR (194 nt)
G1073xxGxGKS Walker A
K1784GDD
S1752G
D1687ATAFD
nt 6513 (M1)
nt 8258 (K582)
T
Teltowkanal hepe-like virus 36 (TkHLV-36 strain 233-17E/595, OP699136, 8024nt)
ORF1
nt 32 (M1)
nt 530 (A167)
nt 1102 (K357)
nt 3401 (G1124)
nt 4081 (G1349)
nt 4805 (K1592)
nt 5677 (S1883)
nt 5743 (I1904)
nt 5833 (M1)
nt 6228 (E132)
VMethTr (pfam01660)
Hel (pfam01443)
RdRP (pfam00978)
ORF2
ORF3
nt 8024
5'-NTR (31 nt)
3'-NTR (127 nt)
G1124xxGxAKT Walker A
K1755GDD
S1723G
D1662VEEWD
nt 6143 (M1)
nt 7897 (D585)
U
Teltowkanal hepe-like virus 39 (TkHLV-39 strain MR233-17E/503, OP699140, 8483 nt)
ORF1
ORF3
nt 53 (M1)
nt 245 (N65)
nt 949 (Y299)
nt 3497 (S1149)
nt 4207 (T1385)
nt 6091 (Q2013)
nt 6266 (M1)
nt 7546 (L427)
nt 7633 (M1)
nt 7662 (H10)
nt 7809 (L59)
nt 8397 (I255)
nt 8483
VMethTr (pfam01660)
Hel (pfam01443)
RdRP?
ORF2
Ubox (smart00504)
5'-NTR (52 nt)
3'-NTR (70 nt)
G1150xxGxGKT Walker A
K1842GDD
S1810G
D1749FSEHD
C13AIT16-C28GH30LFE33-A36-C47PVS
Cross-brace motif

V

Teltowkanal astro-like virus 1 (TkALV-1 strain MR233-17E/955, OP699151, 6353nt)

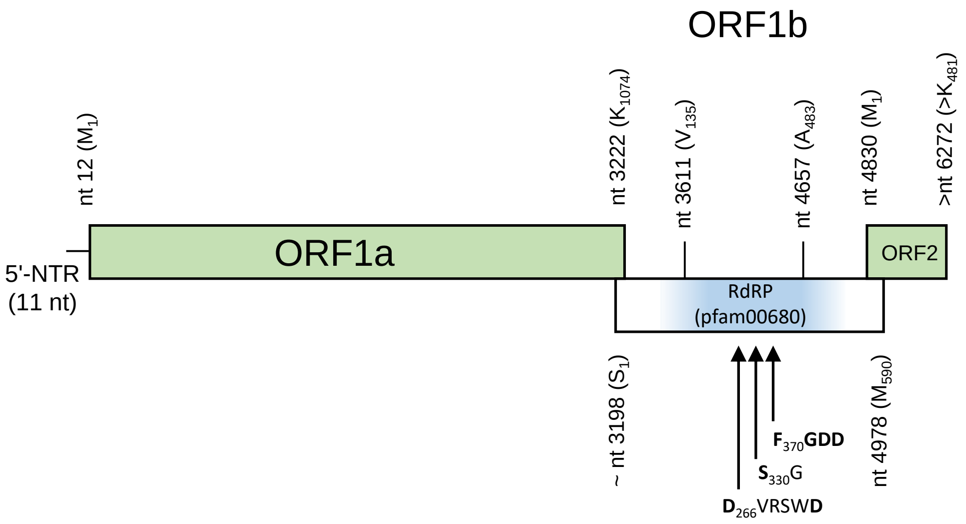

W

Teltowkanal astro-like virus 3 (TkALV-3 strain MR233-17E/832, OP699153, 6815nt)

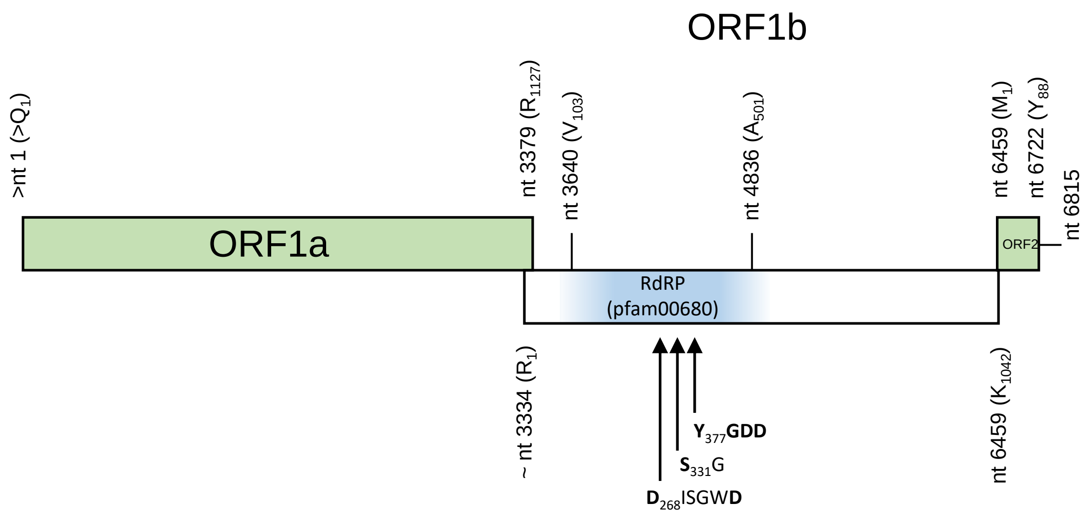

Suppl. Fig. 2A

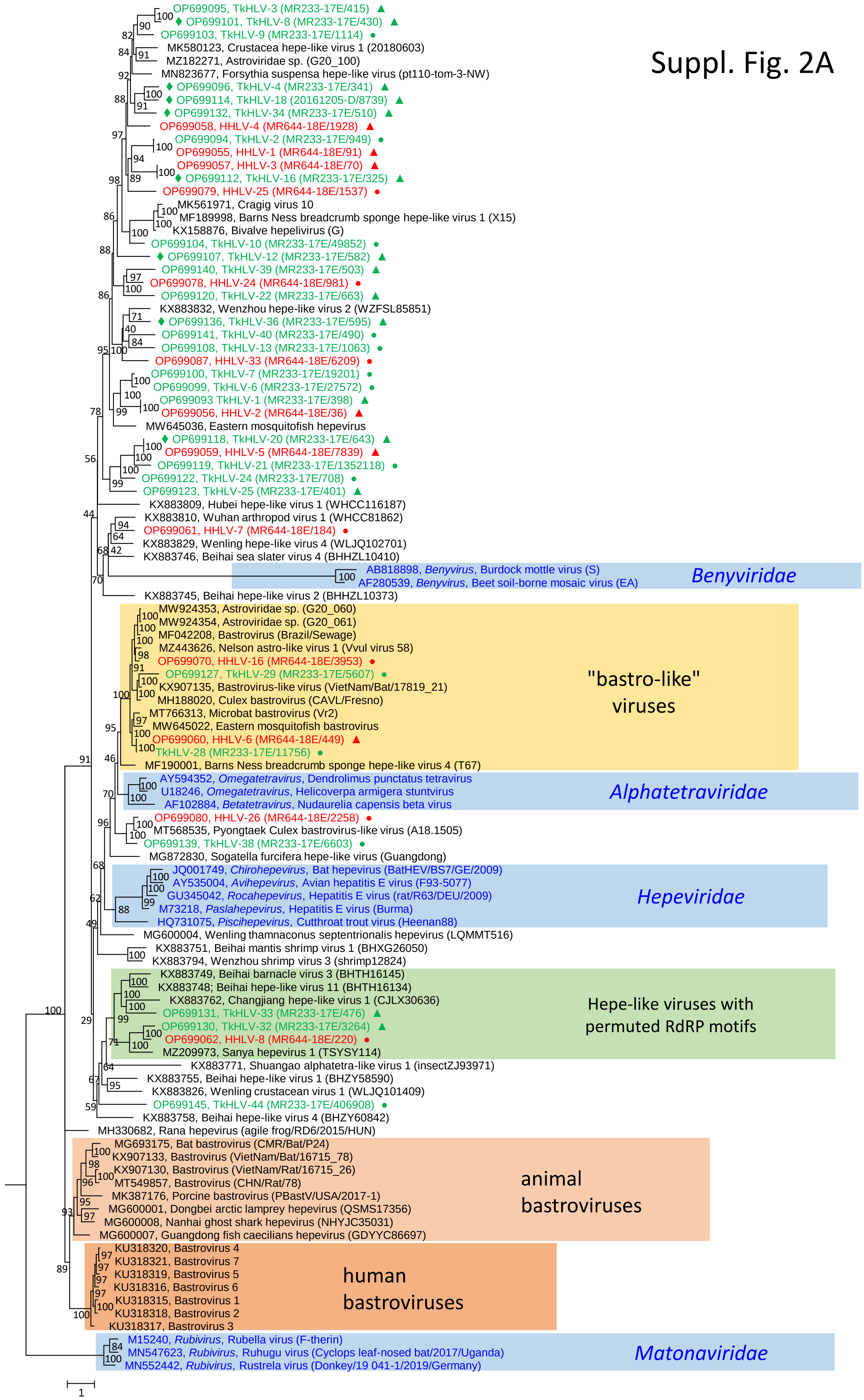

OP699095, TkHLV-3 (MR233-17E/415)
OP699101, TkHLV-8 (MR233-17E/430)
OP699103, TkHLV-9 (MR233-17E/1114)
MK580123, Crustacea hepe-like virus 1 (20180603)
MZ182271, Astroviridae sp. (G20_100)
MN823677, Forsythia suspensa hepe-like virus (pt110-tom-3-NW)
OP699096, TkHLV-4 (MR233-17E/341)
OP699114, TkHLV-18 (20161205-D/8739)
OP699132, TkHLV-34 (MR233-17E/510)
OP699058, HHLV-4 (MR644-18E/1928)
OP699094, TkHLV-2 (MR233-17E/949)
OP699055, HHLV-1 (MR644-18E/91)
OP699057, HHLV-3 (MR644-18E/70)
OP699112, TkHLV-16 (MR233-17E/325)
OP699079, HHLV-25 (MR644-18E/1537)
MK561971, Cragig virus 10
MF189998, Barns Ness breadcrumb sponge hepe-like virus 1 (X15)
KX158876, Bivalve hepelivirus (G)
OP699104, TkHLV-10 (MR233-17E/49852)
OP699107, TkHLV-12 (MR233-17E/582)
OP699140, TkHLV-39 (MR233-17E/503)
OP699078, HHLV-24 (MR644-18E/981)
OP699120, TkHLV-22 (MR233-17E/663)
KX883832, Wenzhou hepe-like virus 2 (WZFSL85851)
OP699136, TkHLV-36 (MR233-17E/595)
OP699141, TkHLV-40 (MR233-17E/490)
OP699108, TkHLV-13 (MR233-17E/1063)
OP699087, HHLV-33 (MR644-18E/6209)
OP699100, TkHLV-7 (MR233-17E/19201)
OP699099, TkHLV-6 (MR233-17E/27572)
OP699093 TkHLV-1 (MR233-17E/398)
OP699056, HHLV-2 (MR644-18E/36)
MW645036, Eastern mosquitofish hepevirus
OP699118, TkHLV-20 (MR233-17E/643)
OP699059, HHLV-5 (MR644-18E/7839)
OP699119, TkHLV-21 (MR233-17E/1352118)
OP699122, TkHLV-24 (MR233-17E/708)
OP699123, TkHLV-25 (MR233-17E/401)
KX883809, Hubei hepe-like virus 1 (WHCC116187)
KX883810, Wuhan arthropod virus 1 (WHCC81862)
OP699061, HHLV-7 (MR644-18E/184)
KX883829, Wenling hepe-like virus 4 (WLJQ102701)
KX883746, Beihai sea slater virus 4 (BHHZL10410)
AB818898, *Benyvirus*, Burdock mottle virus (S)
AF280539, *Benyvirus*, Beet soil-borne mosaic virus (EA)
Benyviridae
KX883745, Beihai hepe-like virus 2 (BHHZL10373)
MW924353, Astroviridae sp. (G20_060)
MW924354, Astroviridae sp. (G20_061)
MF042208, Bastrovirus (Brazil/Sewage)
MZ443626, Nelson astro-like virus 1 (Vvul virus 58)
OP699070, HHLV-16 (MR644-18E/3953)
OP699127, TkHLV-29 (MR233-17E/5607)
KX907135, Bastrovirus-like virus (VietNam/Bat/17819_21)
MH188020, Culex bastrovirus (CAVL/Fresno)
MT766313, Microbat bastrovirus (Vr2)
MW645022, Eastern mosquitofish bastrovirus
OP699060, HHLV-6 (MR644-18E/449)
TkHLV-28 (MR233-17E/11756)
MF190001, Barns Ness breadcrumb sponge hepe-like virus 4 (T67)
"bastro-like" viruses
AY594352, *Omegatetravirus*, Dendrolimus punctatus tetravirus
U18246, *Omegatetravirus*, Helicoverpa armigera stuntvirus
AF102884, *Betatetravirus*, Nudaurelia capensis beta virus
Alphatetraviridae
OP699080, HHLV-26 (MR644-18E/2258)
MT568535, Pyongtaek Culex bastrovirus-like virus (A18.1505)
OP699139, TkHLV-38 (MR233-17E/6603)
MG872830, Sogatella furcifera hepe-like virus (Guangdong)
JQ001749, *Chirohepevirus*, Bat hepevirus (BatHEV/BS7/GE/2009)
AY535004, *Avihepevirus*, Avian hepatitis E virus (F93-5077)
GU345042, *Rocahepevirus*, Hepatitis E virus (rat/R63/DEU/2009)
M73218, *Paslahepevirus*, Hepatitis E virus (Burma)
HQ731075, *Piscihepevirus*, Cutthroat trout virus (Heenan88)
Hepeviridae
MG600004, Wenling thamnaconus septentrionalis hepevirus (LQMMT516)
KX883751, Beihai mantis shrimp virus 1 (BHXG26050)
KX883794, Wenzhou shrimp virus 3 (shrimp12824)
KX883749, Beihai barnacle virus 3 (BHTH16145)
KX883748; Beihai hepe-like virus 11 (BHTH16134)
KX883762, Changjiang hepe-like virus 1 (CJLX30636)
OP699131, TkHLV-33 (MR233-17E/476)
OP699130, TkHLV-32 (MR233-17E/3264)
OP699062, HHLV-8 (MR644-18E/220)
MZ209973, Sanya hepevirus 1 (TSYSY114)
Hepe-like viruses with permuted RdRP motifs
KX883771, Shuangao alphatetra-like virus 1 (insectZJ93971)
KX883755, Beihai hepe-like virus 1 (BHZY58590)
KX883826, Wenling crustacean virus 1 (WLJQ101409)
OP699145, TkHLV-44 (MR233-17E/406908)
KX883758, Beihai hepe-like virus 4 (BHZY60842)
MH330682, Rana hepevirus (agile frog/RD6/2015/HUN)
MG693175, Bat bastrovirus (CMR/Bat/P24)
KX907133, Bastrovirus (VietNam/Bat/16715_78)
KX907130, Bastrovirus (VietNam/Rat/16715_26)
MT549857, Bastrovirus (CHN/Rat/78)
MK387176, Porcine bastrovirus (PBastV/USA/2017-1)
MG600001, Dongbei arctic lamprey hepevirus (QSMS17356)
MG600008, Nanhai ghost shark hepevirus (NHYJC35031)
MG600007, Guangdong fish caecilians hepevirus (GDYYC86697)
animal bastroviruses
KU318320, Bastrovirus 4
KU318321, Bastrovirus 7
KU318319, Bastrovirus 5
KU318316, Bastrovirus 6
KU318315, Bastrovirus 1
KU318318, Bastrovirus 2
KU318317, Bastrovirus 3
human bastroviruses
M15240, *Rubivirus*, Rubella virus (F-therin)
MN547623, *Rubivirus*, Ruhugu virus (Cyclops leaf-nosed bat/2017/Uganda)
MN552442, *Rubivirus*, Rustrela virus (Donkey/19 041-1/2019/Germany)
Matonaviridae
1

Suppl. Fig. 2B

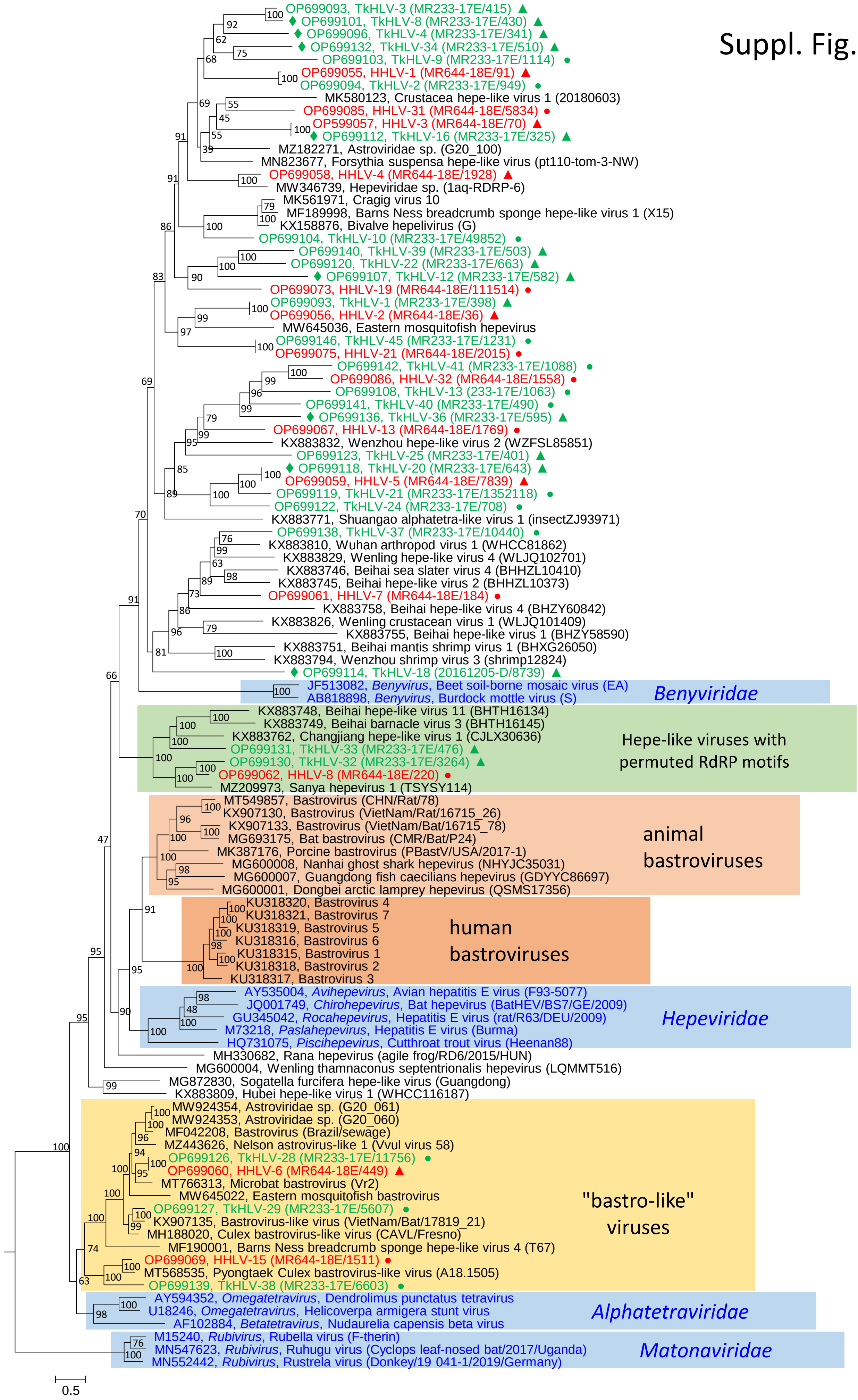

OP699093, TkHLV-3 (MR233-17E/415)
OP699101, TkHLV-8 (MR233-17E/430)
OP699096, TkHLV-4 (MR233-17E/341)
OP699132, TkHLV-34 (MR233-17E/510)
OP699103, TkHLV-9 (MR233-17E/1114)
OP699055, HHLV-1 (MR644-18E/91)
OP699094, TkHLV-2 (MR233-17E/949)
MK580123, Crustacea hepe-like virus 1 (20180603)
OP699085, HHLV-31 (MR644-18E/5834)
OP599057, HHLV-3 (MR644-18E/70)
OP699112, TkHLV-16 (MR233-17E/325)
MZ182271, Astroviridae sp. (G20_100)
MN823677, Forsythia suspensa hepe-like virus (pt110-tom-3-NW)
OP699058, HHLV-4 (MR644-18E/1928)
MW346739, Hepeviridae sp. (1aq-RDRP-6)
MK561971, Cragig virus 10
MF189998, Barns Ness breadcrumb sponge hepe-like virus 1 (X15)
KX158876, Bivalve hepelivirus (G)
OP699104, TkHLV-10 (MR233-17E/49852)
OP699140, TkHLV-39 (MR233-17E/503)
OP699120, TkHLV-22 (MR233-17E/663)
OP699107, TkHLV-12 (MR233-17E/582)
OP699073, HHLV-19 (MR644-18E/111514)
OP699093, TkHLV-1 (MR233-17E/398)
OP699056, HHLV-2 (MR644-18E/36)
MW645036, Eastern mosquitofish hepevirus
OP699146, TkHLV-45 (MR233-17E/1231)
OP699075, HHLV-21 (MR644-18E/2015)
OP699142, TkHLV-41 (MR233-17E/1088)
OP699086, HHLV-32 (MR644-18E/1558)
OP699108, TkHLV-13 (233-17E/1063)
OP699141, TkHLV-40 (MR233-17E/490)
OP699136, TkHLV-36 (MR233-17E/595)
OP699067, HHLV-13 (MR644-18E/1769)
KX883832, Wenzhou hepe-like virus 2 (WZFSL85851)
OP699123, TkHLV-25 (MR233-17E/401)
OP699118, TkHLV-20 (MR233-17E/643)
OP699059, HHLV-5 (MR644-18E/7839)
OP699119, TkHLV-21 (MR233-17E/1352118)
OP699122, TkHLV-24 (MR233-17E/708)
KX883771, Shuangao alphatetra-like virus 1 (insectZJ93971)
OP699138, TkHLV-37 (MR233-17E/10440)
KX883810, Wuhan arthropod virus 1 (WHCC81862)
KX883829, Wenling hepe-like virus 4 (WLJQ102701)
KX883746, Beihai sea slater virus 4 (BHHZL10410)
KX883745, Beihai hepe-like virus 2 (BHHZL10373)
OP699061, HHLV-7 (MR644-18E/184)
KX883758, Beihai hepe-like virus 4 (BHZY60842)
KX883826, Wenling crustacean virus 1 (WLJQ101409)
KX883755, Beihai hepe-like virus 1 (BHZY58590)
KX883751, Beihai mantis shrimp virus 1 (BHXG26050)
KX883794, Wenzhou shrimp virus 3 (shrimp12824)
OP699114, TkHLV-18 (20161205-D/8739)
JF513082, Benyvirus, Beet soil-borne mosaic virus (EA)
AB818898, Benyvirus, Burdock mottle virus (S)
Benyviridae
KX883748, Beihai hepe-like virus 11 (BHTH16134)
KX883749, Beihai barnacle virus 3 (BHTH16145)
KX883762, Changjiang hepe-like virus 1 (CJLX30636)
OP699131, TkHLV-33 (MR233-17E/476)
OP699130, TkHLV-32 (MR233-17E/3264)
OP699062, HHLV-8 (MR644-18E/220)
MZ209973, Sanya hepevirus 1 (TSYSY114)
Hepe-like viruses with permuted RdRP motifs
MT549857, Bastrovirus (CHN/Rat/78)
KX907130, Bastrovirus (VietNam/Rat/16715_26)
KX907133, Bastrovirus (VietNam/Bat/16715_78)
MG693175, Bat bastrovirus (CMR/Bat/P24)
MK387176, Porcine bastrovirus (PBastV/USA/2017-1)
MG600008, Nanhai ghost shark hepevirus (NHYJC35031)
MG600007, Guangdong fish caecilians hepevirus (GDYYC86697)
MG600001, Dongbei arctic lamprey hepevirus (QSMS17356)
animal bastroviruses
KU318320, Bastrovirus 4
KU318321, Bastrovirus 7
KU318319, Bastrovirus 5
KU318316, Bastrovirus 6
KU318315, Bastrovirus 1
KU318318, Bastrovirus 2
KU318317, Bastrovirus 3
human bastroviruses
AY535004, Avihepevirus, Avian hepatitis E virus (F93-5077)
JQ001749, Chirohepevirus, Bat hepevirus (BatHEV/BS7/GE/2009)
GU345042, Rocahepevirus, Hepatitis E virus (rat/R63/DEU/2009)
M73218, Paslahepevirus, Hepatitis E virus (Burma)
HQ731075, Piscihepevirus, Cutthroat trout virus (Heenan88)
Hepeviridae
MH330682, Rana hepevirus (agile frog/RD6/2015/HUN)
MG600004, Wenling thamnaconus septentrionalis hepevirus (LQMMT516)
MG872830, Sogatella furcifera hepe-like virus (Guangdong)
KX883809, Hubei hepe-like virus 1 (WHCC116187)
MW924354, Astroviridae sp. (G20_061)
MW924353, Astroviridae sp. (G20_060)
MF042208, Bastrovirus (Brazil/sewage)
MZ443626, Nelson astrovirus-like 1 (Vvul virus 58)
OP699126, TkHLV-28 (MR233-17E/11756)
OP699060, HHLV-6 (MR644-18E/449)
MT766313, Microbat bastrovirus (Vr2)
MW645022, Eastern mosquitofish bastrovirus
OP699127, TkHLV-29 (MR233-17E/5607)
KX907135, Bastrovirus-like virus (VietNam/Bat/17819_21)
MH188020, Culex bastrovirus-like virus (CAVL/Fresno)
MF190001, Barns Ness breadcrumb sponge hepe-like virus 4 (T67)
OP699069, HHLV-15 (MR644-18E/1511)
MT568535, Pyongtaek Culex bastrovirus-like virus (A18.1505)
OP699139, TkHLV-38 (MR233-17E/6603)
"bastro-like" viruses
AY594352, Omegatetravirus, Dendrolimus punctatus tetravirus
U18246, Omegatetravirus, Helicoverpa armigera stunt virus
AF102884, Betatetravirus, Nudaurelia capensis beta virus
Alphatetraviridae
M15240, Rubivirus, Rubella virus (F-therin)
MN547623, Rubivirus, Ruhugu virus (Cyclops leaf-nosed bat/2017/Uganda)
MN552442, Rubivirus, Rustrela virus (Donkey/19 041-1/2019/Germany)
Matonaviridae
0.5

Suppl. Fig. 2C

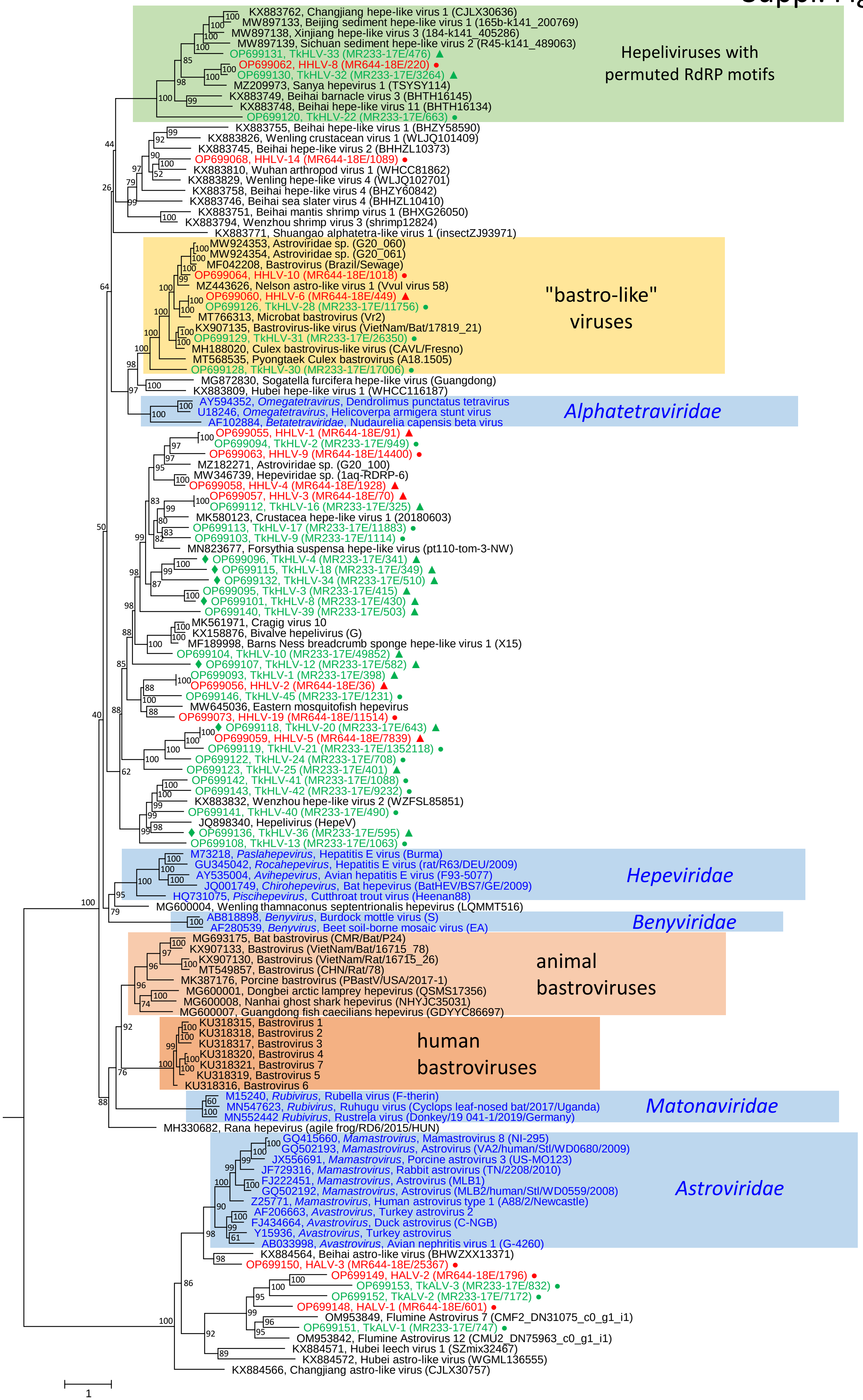
KX883762, Changjiang hepe-like virus 1 (CJLX30636)
MW897133, Beijing sediment hepe-like virus 1 (165b-k141_200769)
MW897138, Xinjiang hepe-like virus 3 (184-k141_405286)
MW897139, Sichuan sediment hepe-like virus 2 (R45-k141_489063)
OP699131, TkHLV-33 (MR233-17E/476)
OP699062, HHLV-8 (MR644-18E/220)
OP699130, TkHLV-32 (MR233-17E/3264)
MZ209973, Sanya hepevirus 1 (TSYSY114)
KX883749, Beihai barnacle virus 3 (BHTH16145)
KX883748, Beihai hepe-like virus 11 (BHTH16134)
OP699120, TkHLV-22 (MR233-17E/663)
Hepeliviruses with permuted RdRP motifs
KX883755, Beihai hepe-like virus 1 (BHZY58590)
KX883826, Wenling crustacean virus 1 (WLJQ101409)
KX883745, Beihai hepe-like virus 2 (BHHZL10373)
OP699068, HHLV-14 (MR644-18E/1089)
KX883810, Wuhan arthropod virus 1 (WHCC81862)
KX883829, Wenling hepe-like virus 4 (WLJQ102701)
KX883758, Beihai hepe-like virus 4 (BHZY60842)
KX883746, Beihai sea slater virus 4 (BHHZL10410)
KX883751, Beihai mantis shrimp virus 1 (BHXG26050)
KX883794, Wenzhou shrimp virus 3 (shrimp12824)
KX883771, Shuangao alphatetra-like virus 1 (insectZJ93971)
MW924353, Astroviridae sp. (G20_060)
MW924354, Astroviridae sp. (G20_061)
MF042208, Bastrovirus (Brazil/Sewage)
OP699064, HHLV-10 (MR644-18E/1018)
MZ443626, Nelson astro-like virus 1 (Vvul virus 58)
OP699060, HHLV-6 (MR644-18E/449)
OP699126, TkHLV-28 (MR233-17E/11756)
MT766313, Microbat bastrovirus (Vr2)
KX907135, Bastrovirus-like virus (VietNam/Bat/17819_21)
OP699129, TkHLV-31 (MR233-17E/26350)
MH188020, Culex bastrovirus-like virus (CAVL/Fresno)
MT568535, Pyongtaek Culex bastrovirus (A18.1505)
OP699128, TkHLV-30 (MR233-17E/17006)
"bastro-like" viruses
MG872830, Sogatella furcifera hepe-like virus (Guangdong)
KX883809, Hubei hepe-like virus 1 (WHCC116187)
AY594352, Omegatetravirus, Dendrolimus punctatus tetravirus
U18246, Omegatetravirus, Helicoverpa armigera stunt virus
AF102884, Betatetravirus, Nudaurelia capensis beta virus
Alphatetraviridae
OP699055, HHLV-1 (MR644-18E/91)
OP699094, TkHLV-2 (MR233-17E/949)
OP699063, HHLV-9 (MR644-18E/14400)
MZ182271, Astroviridae sp. (G20_100)
MW346739, Hepeviridae sp. (1aq-RDRP-6)
OP699058, HHLV-4 (MR644-18E/1928)
OP699057, HHLV-3 (MR644-18E/70)
OP699112, TkHLV-16 (MR233-17E/325)
MK580123, Crustacea hepe-like virus 1 (20180603)
OP699113, TkHLV-17 (MR233-17E/11883)
OP699103, TkHLV-9 (MR233-17E/1114)
MN823677, Forsythia suspensa hepe-like virus (pt110-tom-3-NW)
OP699096, TkHLV-4 (MR233-17E/341)
OP699115, TkHLV-18 (MR233-17E/349)
OP699132, TkHLV-34 (MR233-17E/510)
OP699095, TkHLV-3 (MR233-17E/415)
OP699101, TkHLV-8 (MR233-17E/430)
OP699140, TkHLV-39 (MR233-17E/503)
MK561971, Cragig virus 10
KX158876, Bivalve hepelivirus (G)
MF189998, Barns Ness breadcrumb sponge hepe-like virus 1 (X15)
OP699104, TkHLV-10 (MR233-17E/49852)
OP699107, TkHLV-12 (MR233-17E/582)
OP699093, TkHLV-1 (MR233-17E/398)
OP699056, HHLV-2 (MR644-18E/36)
OP699146, TkHLV-45 (MR233-17E/1231)
MW645036, Eastern mosquitofish hepevirus
OP699073, HHLV-19 (MR644-18E/11514)
OP699118, TkHLV-20 (MR233-17E/643)
OP699059, HHLV-5 (MR644-18E/7839)
OP699119, TkHLV-21 (MR233-17E/1352118)
OP699122, TkHLV-24 (MR233-17E/708)
OP699123, TkHLV-25 (MR233-17E/401)
OP699142, TkHLV-41 (MR233-17E/1088)
OP699143, TkHLV-42 (MR233-17E/9232)
KX883832, Wenzhou hepe-like virus 2 (WZFSL85851)
OP699141, TkHLV-40 (MR233-17E/490)
JQ898340, Hepelivirus (HepeV)
OP699136, TkHLV-36 (MR233-17E/595)
OP699108, TkHLV-13 (MR233-17E/1063)
M73218, Paslahepevirus, Hepatitis E virus (Burma)
GU345042, Rocahepevirus, Hepatitis E virus (rat/R63/DEU/2009)
AY535004, Avihepevirus, Avian hepatitis E virus (F93-5077)
JQ001749, Chirohepevirus, Bat hepevirus (BatHEV/BS7/GE/2009)
HQ731075, Piscihepevirus, Cutthroat trout virus (Heenan88)
Hepeviridae
MG600004, Wenling thamnaconus septentrionalis hepevirus (LQMMT516)
AB818898, Benyvirus, Burdock mottle virus (S)
AF280539, Benyvirus, Beet soil-borne mosaic virus (EA)
Benyviridae
MG693175, Bat bastrovirus (CMR/Bat/P24)
KX907133, Bastrovirus (VietNam/Bat/16715_78)
KX907130, Bastrovirus (VietNam/Rat/16715_26)
MT549857, Bastrovirus (CHN/Rat/78)
MK387176, Porcine bastrovirus (PBastV/USA/2017-1)
MG600001, Dongbei arctic lamprey hepevirus (QSMS17356)
MG600008, Nanhai ghost shark hepevirus (NHYJC35031)
MG600007, Guangdong fish caecilians hepevirus (GDYYC86697)
animal bastroviruses
KU318315, Bastrovirus 1
KU318318, Bastrovirus 2
KU318317, Bastrovirus 3
KU318320, Bastrovirus 4
KU318321, Bastrovirus 7
KU318319, Bastrovirus 5
KU318316, Bastrovirus 6
human bastroviruses
M15240, Rubivirus, Rubella virus (F-therin)
MN547623, Rubivirus, Ruhugu virus (Cyclops leaf-nosed bat/2017/Uganda)
MN552442 Rubivirus, Rustrela virus (Donkey/19 041-1/2019/Germany)
Matonaviridae
MH330682, Rana hepevirus (agile frog/RD6/2015/HUN)
GQ415660, Mamastrovirus, Mamastrovirus 8 (NI-295)
GQ502193, Mamastrovirus, Astrovirus (VA2/human/Stl/WD0680/2009)
JX556691, Mamastrovirus, Porcine astrovirus 3 (US-MO123)
JF729316, Mamastrovirus, Rabbit astrovirus (TN/2208/2010)
FJ222451, Mamastrovirus, Astrovirus (MLB1)
GQ502192, Mamastrovirus, Astrovirus (MLB2/human/Stl/WD0559/2008)
Z25771, Mamastrovirus, Human astrovirus type 1 (A88/2/Newcastle)
AF206663, Avastrovirus, Turkey astrovirus 2
FJ434664, Avastrovirus, Duck astrovirus (C-NGB)
Y15936, Avastrovirus, Turkey astrovirus
AB033998, Avastrovirus, Avian nephritis virus 1 (G-4260)
Astroviridae
KX884564, Beihai astro-like virus (BHWZXX13371)
OP699150, HALV-3 (MR644-18E/25367)
OP699149, HALV-2 (MR644-18E/1796)
OP699153, TkALV-3 (MR233-17E/832)
OP699152, TkALV-2 (MR233-17E/7172)
OP699148, HALV-1 (MR644-18E/601)
OM953849, Flumine Astrovirus 7 (CMF2_DN31075_c0_g1_i1)
OP699151, TkALV-1 (MR233-17E/747)
OM953842, Flumine Astrovirus 12 (CMU2_DN75963_c0_g1_i1)
KX884571, Hubei leech virus 1 (SZmix32467)
KX884572, Hubei astro-like virus (WGML136555)
KX884566, Changjiang astro-like virus (CJLX30757)
1

Suppl. Fig. 3

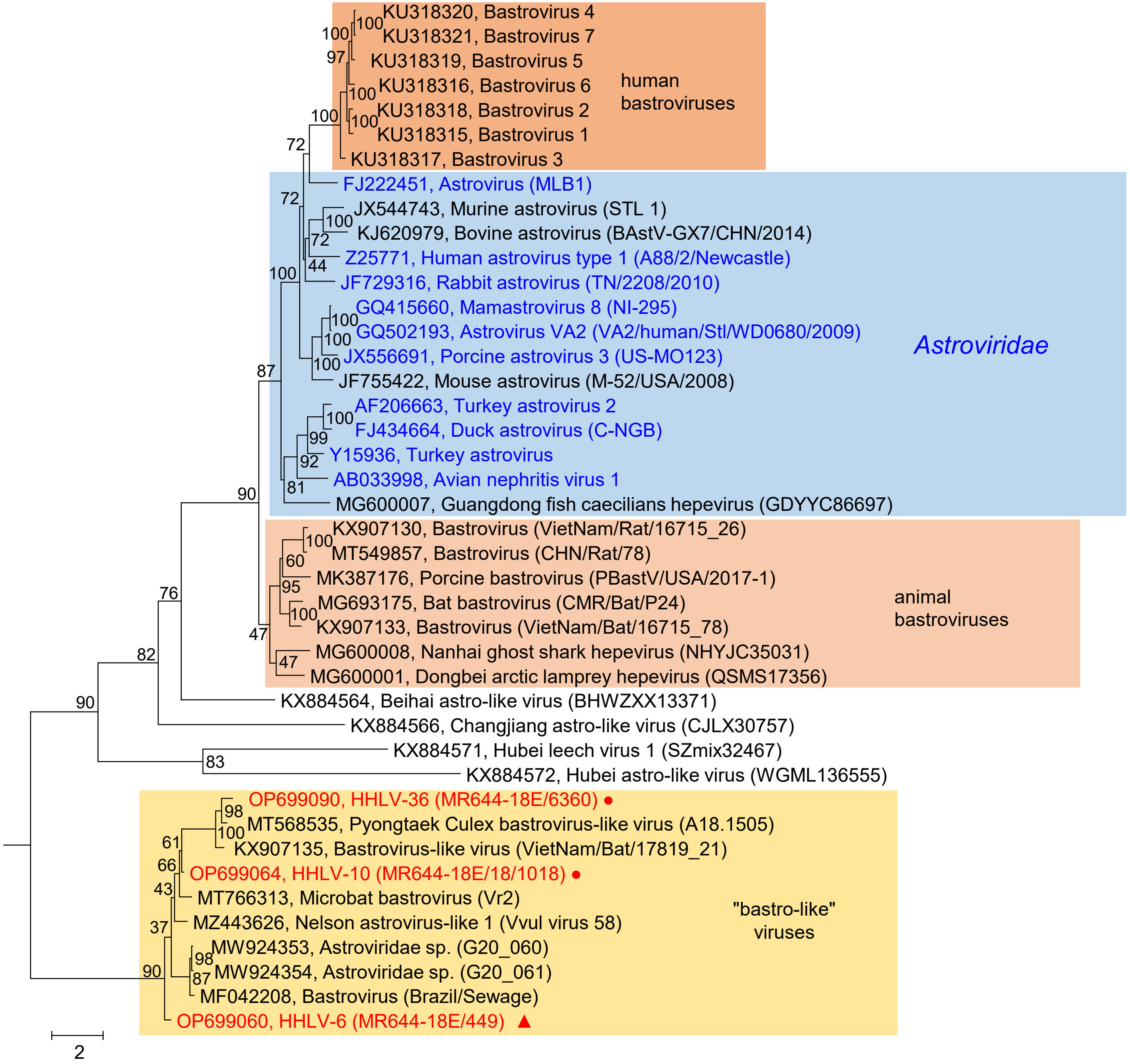
KU318320, Bastrovirus 4
KU318321, Bastrovirus 7
KU318319, Bastrovirus 5
KU318316, Bastrovirus 6
KU318318, Bastrovirus 2
KU318315, Bastrovirus 1
KU318317, Bastrovirus 3
human bastroviruses
FJ222451, Astrovirus (MLB1)
JX544743, Murine astrovirus (STL 1)
KJ620979, Bovine astrovirus (BAstV-GX7/CHN/2014)
Z25771, Human astrovirus type 1 (A88/2/Newcastle)
JF729316, Rabbit astrovirus (TN/2208/2010)
GQ415660, Mamastrovirus 8 (NI-295)
GQ502193, Astrovirus VA2 (VA2/human/Stl/WD0680/2009)
JX556691, Porcine astrovirus 3 (US-MO123)
JF755422, Mouse astrovirus (M-52/USA/2008)
AF206663, Turkey astrovirus 2
FJ434664, Duck astrovirus (C-NGB)
Y15936, Turkey astrovirus
AB033998, Avian nephritis virus 1
MG600007, Guangdong fish caecilians hepevirus (GDYYC86697)
Astroviridae
KX907130, Bastrovirus (VietNam/Rat/16715_26)
MT549857, Bastrovirus (CHN/Rat/78)
MK387176, Porcine bastrovirus (PBastV/USA/2017-1)
MG693175, Bat bastrovirus (CMR/Bat/P24)
KX907133, Bastrovirus (VietNam/Bat/16715_78)
MG600008, Nanhai ghost shark hepevirus (NHYJC35031)
MG600001, Dongbei arctic lamprey hepevirus (QSMS17356)
animal bastroviruses
KX884564, Beihai astro-like virus (BHWZXX13371)
KX884566, Changjiang astro-like virus (CJLX30757)
KX884571, Hubei leech virus 1 (SZmix32467)
KX884572, Hubei astro-like virus (WGML136555)
OP699090, HHLV-36 (MR644-18E/6360) ●
MT568535, Pyongtaek Culex bastrovirus-like virus (A18.1505)
KX907135, Bastrovirus-like virus (VietNam/Bat/17819_21)
OP699064, HHLV-10 (MR644-18E/18/1018) ●
MT766313, Microbat bastrovirus (Vr2)
MZ443626, Nelson astrovirus-like 1 (Vvul virus 58)
MW924353, Astroviridae sp. (G20_060)
MW924354, Astroviridae sp. (G20_061)
MF042208, Bastrovirus (Brazil/Sewage)
OP699060, HHLV-6 (MR644-18E/449) ▲
"bastro-like" viruses
100
100
97
100
100
100
72
72
100
72
44
100
100
100
100
87
100
99
92
81
90
100
60
95
100
47
47
76
82
90
83
98
100
61
66
43
37
98
87
90
2

Suppl. Fig. 4

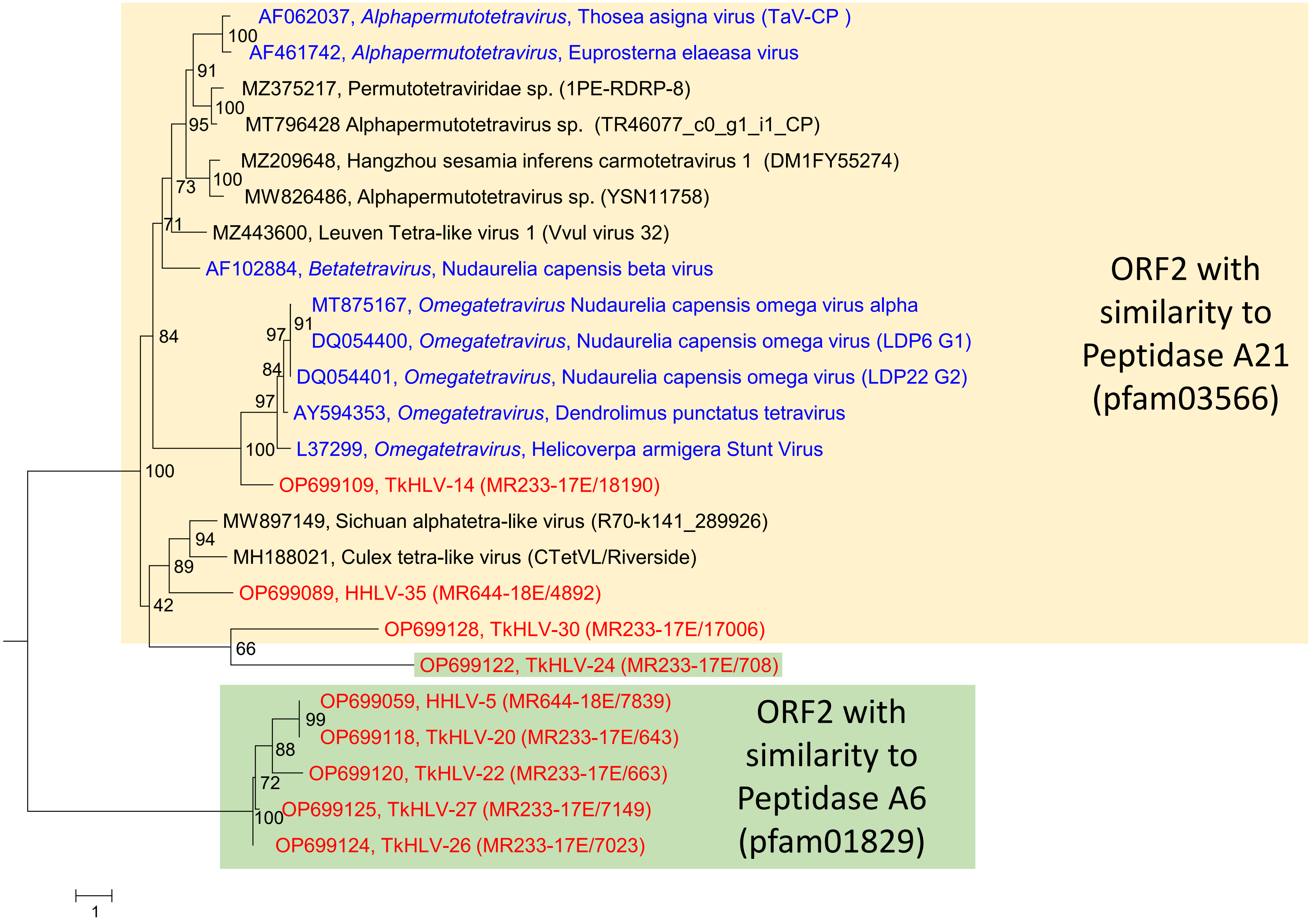
AF062037, *Alphapermutotetravirus*, Thosea asigna virus (TaV-CP)
AF461742, *Alphapermutotetravirus*, Euprosterna elaeasa virus
MZ375217, Permutotetraviridae sp. (1PE-RDRP-8)
MT796428 Alphapermutotetravirus sp. (TR46077_c0_g1_i1_CP)
MZ209648, Hangzhou sesamia inferens carmotetravirus 1 (DM1FY55274)
MW826486, Alphapermutotetravirus sp. (YSN11758)
MZ443600, Leuven Tetra-like virus 1 (Vvul virus 32)
AF102884, *Betatetravirus*, Nudaurelia capensis beta virus
MT875167, *Omegatetravirus* Nudaurelia capensis omega virus alpha
DQ054400, *Omegatetravirus*, Nudaurelia capensis omega virus (LDP6 G1)
DQ054401, *Omegatetravirus*, Nudaurelia capensis omega virus (LDP22 G2)
AY594353, *Omegatetravirus*, Dendrolimus punctatus tetravirus
L37299, *Omegatetravirus*, Helicoverpa armigera Stunt Virus
OP699109, TkHLV-14 (MR233-17E/18190)
MW897149, Sichuan alphatetra-like virus (R70-k141_289926)
MH188021, Culex tetra-like virus (CTetVL/Riverside)
OP699089, HHLV-35 (MR644-18E/4892)
OP699128, TkHLV-30 (MR233-17E/17006)
OP699122, TkHLV-24 (MR233-17E/708)
OP699059, HHLV-5 (MR644-18E/7839)
OP699118, TkHLV-20 (MR233-17E/643)
OP699120, TkHLV-22 (MR233-17E/663)
OP699125, TkHLV-27 (MR233-17E/7149)
OP699124, TkHLV-26 (MR233-17E/7023)
100
91
100
95
100
73
71
84
91
97
84
97
100
100
94
89
42
66
99
88
72
100
1
ORF2 with similarity to Peptidase A21 (pfam03566)
ORF2 with similarity to Peptidase A6 (pfam01829)

Supplementary Figure 5

	permuted motif C (spanning the 6th–8th blocks); motif A (spanning the end of the 8th and the 9th block)
TkHLV-4 MR233-17E/341	KILNVIMSTF CKGFESDFKN HLSDK---IL LAIDESDKDL SVEFAKRGHL YVD------- ---------- --NKYRRLDC DITEFDSSQN AKTVAMHCLI
TkHLV-34 MR233-17E/510	KISNIMFASM QKQLHDWVKK SLADN---CM WAVDKSDHDI AEEFKKKGYG AHL------- ---------- TDARAKTHSK DATAFDQSQK HVGNVAIAVL
MW346739 Hepeviridae sp. 1aq-RDRP-6	KMYNVMISGM VRYFSDIMNS IDKDF---VQ NSYNMSDKDL SNKFKKCVGH TIRN------ ---------- --RNIKKFAN DFSEFDSAQD EISILAFAAF
HHLV-3 MR644-18//1928	KMYNIMISGM IRYFSDIMNS IDLDY---VQ NSYNESDRDL SVKFKKCAGH TIKN------ ---------- --NNIKKLVN DFKQFDSAQD ETSILAFASF
HHLV-9 MR644-18E/14400	KLANLVNAAY IRYFDSLLPF LIRDN---VQ LSYNESDKDL STFWMRFASS LND------- ---------- --PTVIKFLN DFGQFDSTQE KRGVMGISMM
HHLV-1 MR644-18E/91	KMVNVILSGY VRYFSAIVPR ILRDN---VH LAYGYSDKDI SALFSKYSRE IGD------- ---------- --RRYHKFCN DFGEFDASQE QKGLHVLIAF
TkHLV-2 MR233-17E/949	KMVNVILSGY VRYFSAIIPR ILRDN---VH LAYGYSDKDI SALFSKYSRE IGD------- ---------- --RRYHKFCN DFGEFDASQE QKGLHVLIAF
TkHLV-17 MR233-17E/11883	KLLNCIFSGF CRFYSANIKD CLKPN---VQ LAYGASDADL SIFFRKYAKD LND------- ---------- --TNKTKFMA DISEFDSSQE EKAILASATM
HHLV-4 MR644-18E/70	KLLNVVMSGM TRTYDELIRK YVKDN---VN LSYGKSDADI AEFTNDYAKQ LNS------- ---------- --KSYLKVMA DFSEFDSSQE EQGILASIEI
MN823677 Forsythia suspensa hepe-like virus pt110-tom-3-NW	KLLNIIFSSM TRTYARFITK YLKPN---VQ LAYGKADVLI AEDFKKYSDY INS------- ---------- --KNYQKLTC DFSEFDSSQE KQGILSSCMI
MK580123 Crustacea hepe-like virus 1 20180603	KLINIVVSSC TRWYTLHIKD VIKDN---VQ IASGKSDREL AEFFAPLAPK LNA------- ---------- --VAKTKLMA DFSEFDCSQE EKGIVASVAV
MF189998 Barns Ness breadcrumb sponge hepe-like virus 1 X15	KVLNLFFCAY SRYLTECIFE CMNEN---VL LAFNKSDAEL SVFFAGYKDY YTS------- ---------- --EKYVNCNC DFSEMDVSHT KSMLELELEL
MK561971 Cragig virus 10	KVLNLFFCAY SRYLTECIFK SAKPN---VL LAFNKSDAEL STFFAQYKDQ YTS------- ---------- --EKYSNVNC DFSEMDTSHT KSMLELELEL
TkHLV-1 MR233-17E/398	KLLNLVFAAF ARFYSARLKN FLKPG---VF FQNGAPDAAM GDEASLFLMN YYKK------ ---------- TGKLPKFADN DFTEFDASQN EMTIEYDCDH
HHLV-2_MR2018E/36	KLLNLVFAAF ARFYSARLKN FLKPG---VF FQNGAPDAAM GDEASLFLMN YYKK------ ---------- TGKLPKFADN DFTEFDASQN EMTIEYDCDH
HHLV-10 MR644-18E/1018	KTLNILMAPW TRLLEQVLVN QSNGR---VK ILSQKSDLEV MSIIEKDTTE G--------- ---------- ----ERFVDN DWTQFDSNQN NLTREILRRA
HHLV-6 MR644-18E/449	KTLNLLMSPW TRLLEQILVN QSKGR---VH ILSQMSDHMV MSILEKHSED G--------- ---------- ----ERFIGN DWTQFDSNQN NLTRQILRLA
MH188020 Culex bastrovirus-like virus CAVL/Fresno	KTLNLIMAPW TRLLEQVLVN QSKGT---VR ILSQMSDTQV MAILLKDTVE G--------- ---------- ----ERYIDN DWTKFDSNQN NLTREILKRA
KX907135 Bastrovirus-like virus VietNam/Bat/17819_21	KTLNLIMAPW TRLLEQVLVN QSKGT---VR ILSQMSDSQV MAILEQDTVE G--------- ---------- ----EKYIDN DWTKFDSNQN NLTREILRRA
TkHLV-31 MR233-17E/5834	KTLNLIMAPW TRLLEQVLVN QSKGT---VR ILSQMSDTQV MAILEKDTVE G--------- ---------- ----EKYIDN DWTKFDSNQN NLTREILRRA
MT568535 Pyongtaek Culex bastrovirus A18.1505	KNLNFEMMIY CRLLEHVLTR QSRGR---VI LATGMTDLQV LTLLEKLHQP N--------- ---------- ----DQSFSN DWTEFDSSQN VVGRKLLARA
TkHLV-30 MR233-17E/17006	KSLNFMMIIW TRLLERVALR ATNG----FI FASNRTDTEM LQLLDQISQE D--------- ---------- ----HEYLEC DWTEFDSSQN NLEHHLLMCN
KU318315 Bastrovirus 1	HLIN?FFSQV FRAAAAALRA SFKDN---VL FVDGHSEKQL SAFVARHYDW R--------- ---------- ----DTYLAN DFTAFDSTQG ERTMLLEVEL
KU318318 Bastrovirus 2	HLVNLFFSQV FRAAAAALRA SFKDN---VL FVDGHSEKQL SAFVARHYDW R--------- ---------- ----DTYLAN DFTAFDSTQG ERTMLLEVEL
KU318317 Bastrovirus 3	HLINLFFSQV FRAVAAALKR SFKDN---VL FVDGHSEADL SAFVARHYDW R--------- ---------- ----DTYIAN DFTAFDSTQG PRTMDLEVEI
MK387176 Porcine bastrovirus PBastV/USA/2017-1	KTANMTVAPF IRAAQEIVCR GLPDN---IL LVLHQSDQDL KDWFEQHALP ---------- ---------- ----TGAWCN DFTEFDSTQN AGTLDFECQL
MG693175 Bat bastrovirus CMR/Bat/P24	KKANMVVGPF VRAAQERLLR RLKPT---VL MILYQSDADV RDWIRTVNTG ---------- ---------- ----ETCVCN DFTEFDSTQN NGTTEFECLL
MT549857 Bastrovirus CHN/Rat/78	KQANLAIGPF VRAAQEVLLR CLKPN---VC MVLYQSERQL ADWINEQDLE ---------- ---------- ----QPCVCN DFTEFDSTQN DTTAHFEAKV
AY594352, *Omegatetravirus*, Dendrolimus punctatus tetravirus	KSLNFVLAVW VRILDEVLRT GSTT----VR YSNGLPDEEE AMLLEAAVNK VPN------- ---------- ----ASFTSA DWTEFDTAHN NVSEIFFAEL
U18246, *Omegatetravirus*, Helicoverpa armigera stunt virus	KSLNFVLAAW IRILEEILRT GSRT----VR YSNGLPDEEE AMLLEAKINQ VPH------- ---------- ----ATFVSA DWTEFDTAHN NTSELLFAAL
AF102884, *Betatetravirus*, Nudaurelia capensis beta virus	KCANIALSAW VRIIQDQMST --SEK---FI FANGQSDRDT MSIIEARLQE KA-------- ---------- ----REFKSI DIKEFDTVHN WVSILVFSWR
M73218, *Paslahepevirus*, Hepatitis E virus Burma	KTFCALFGPW FRAIEKAILA LLPQG---VF YGDAFDDTVF SAAVAAAKAS ---------- ---------- ----MVF-EN DFSEFDSTQN NFSLGLECAI
GU345042, *Rocahepevirus*, Hepatitis E virus rat/R63/DEU/2009	KTFVALFGPW FRAIEKAIVD TLPEW---CF YGDCYTQEKL EAAVAGAKAC ---------- ---------- ----RVF-EN DFSEFDSTQN NYSLGLECLL
AY535004, *Avihepevirus*, Avian hepatitis E virus F93-5077	KTLCALFGPW FRAIEKHLVA GLPPG---YY YGDLYTEADL HRSVLCAPAG ---------- ---------- ---HLVF-EN DFSEFDSTQN NVSLDLECEL
JQ001749, *Chirohepevirus*, Bat hepevirus BatHEV/BS7/GE/2009	KTLCALFGPW FRAIEKRIVA QLPSG---WF YGDLYTEADL HAHAMAVPDG ---------- ---------- ---IKVF-EN DFSEFDSTQN NVSLSLECEL
HQ731075, *Piscihepevirus*, Cutthroat trout virus Heenan88	KTLCFIYGLF FRKLEKELRQ ALKPS---II FSNPYTEEQL ARAVSKCTTG ---------- ---------- ---TTVFYEN DFSAFDSTQN LVSLGNEFLI
M15240, *Rubivirus*, Rubella virus F-therin	KEWVQVMSPH FRAIQKIIMR ALRPQ---FL VAAGHTEPEV DAWWQAHY-- ---------- ---------- ---TTNAIEV DFTEFDMNQT LATRDVELEI
MN547623, *Rubivirus*, Ruhugu virus Cyclops leaf-nosed bat/2017/Uganda	KDWVMTMSPH FRSIQKIILK AMRDQ---FL VAAGHTAAEI DIWWQANY-- ---------- ---------- ---SPAAVEV DFTEFDMNQT LATRDVEAEI
MN552442, *Rubivirus*, Rustrela virus Donkey/19_041-1/2019/Germany	KDWVVAMSPH FRAIQGILLR ALREE---FC IAAGRTETEL DAWWQAYY-- ---------- ---------- ---SPHAVEV DFTEFDTNQS LATRDVELEI
TkHLV-22 MR233-17E/663	KRVNLIFAAY TRHLMLKLID MPAADGSDLI IGVGRSDREI GMRISDALYH HKN-IFAFKG DDTYIKWM-- -- -NKQLAT DFEQHDTSHS LVVKMWFCYD
HHLV-8 MR644-18E/220	KPLCFVFASW INAIEERYLK ELRPW---VV YATQLGEDEL VGRINSVLR- KYHKCVLVKG DDVSIVFMDE N-SEMRAISW DAAKFDSTQA AVVMAAQALV
TkHLV-32 MR233-17E/3264	KPLCFVFASW INAIEERYLK ELRPW---VV YATQMGEAEM VGRINNILR- NYHKCVLVKG DDVSIIYMDE N-SKMRAISW DAVKFDSTQA AVVMACQALV
MZ209973 Sanya hepevirus 1 TSYSY114	KPLCYVYAAY IGAIEKRWET ELLPY---VI YATRMNDNEL VATINTTLK- RFRKAILIKG DDVAIIFEKA --GKMACICW DASKFDSTQA TAVMVMQAYE
TkHLV-33 MR233-17E/476	PATNVVFGGF VAALEKRRQE ELQPW---VF HATGMSNSDM QHHMEQIMQ- RYGILVAFIG DDVLIAVQQA D-GSWMHFWS DISRFDASYA EIHYGLEVAN
KX883762 Changjiang hepe-like virus 1 CJLX30636	KARNLMLGAY LNAIEKRQQH MLKDF---VL HATGYSDEDL ARTLEERMQ- RNGLILAIKG DDVIAGKRDN D-GHWSYYES DQSQFDASFS TLHYAYEAEW
KX883749 Beihai barnacle virus 3 BHTH16145	KIWNIVSAPY AKVMQKRLCD DTHPW---FM VASGYSEEDM ARKMSQSVS- NHGCFFGTKG DDAVMILLK- D-GEYHVHSC DQTQFDSRQS TLHFSLQDEF
KX883748 Beihai hepe-like virus 11 BHTH16134	KFMNLLEYPH VMHLMAELER CLKPW---VL IATGMSDEEL VLKMENLHT- AHNVVFIIKG DDFLASTRDI QTGRWAFHTL DGKQFDKHIE NVAFDVERRF
MW897133 Beijing sediment hepe-like virus 1165b-k141_200769	KARNLLLGAY LNAIEKRQQD MLKDW---VV HATGYSDEDL ARTLEDRMQ- RNGILLAIKG DDVIAGKRDE T GHWSYFES DQSQFDASFS TLHYAYEAEW
MW897139 Sichuan sediment hepe-like virus 2 R45-k141_489063	KARNLLMGCW INAIEEVETN TLQPW---VL HATGMEDSEI CARMEEIMT- TYGFCICIKG DDTFVAALVN --GEWKYYCS DQSQFDTSFS SVHYTLESCR
MW897138 Xinjiang hepe-like virus 3 184-k141_405286	KARNLLMGAY LHAMERREIK TLKPW---VI HATGFSDTDL CTEMEARME- AHGLILAIKG DDVVVGKRET D GAWTYFES DQSQFDASFS GLHYSLEALR

Supplementary Figure 5 (continued)

motif B — canonical motif C

Sequence	Alignment
TkHLV-4 MR233-17E/341	LSQQGFNSKA IGFMANMRSD YRCNALINS- KLVQRIKFDV SYVMTSGALF TLSGNTIVNM AIIGASFDFT NLQFGAFKGD DSHV--VAGG IKTIKHGNML
TkHLV-34 MR233-17E/510	AKSAGAPART VDHHVNQREK WTATAMFDVD QRVQHALFVS RYMMTSGHLV TLTFNTMYSM TIIGGGFKYT NLRFAMFKGD DSVV--CCDK CEERKYDGQT
MW346739 Hepeviridae sp. 1aq-RDRP-6	LSMLGFNEKC IKLYVNLRLK WVMALRGSSL QHDTITTLKG VFKKHSGEPA TLCGNTWFNR AVTCLCVTFL NPYICAFKGD DSLI--VADS VTVVRSGVDY
HHLV-3 MR644-18//1928	LRMLKMPEKC VKLYTKLRLK WVMALRGSNL QQDTITLLRG VFKKHSGEPA TLDGNTWFNR AVTCMCITWK NPYIAGFKGD DSII--IADE ITVERHGKDS
HHLV-9 MR644-18E/14400	YRACNVSEFT IQFMEKMRQS WTLRMRISGK TMMASALLQG HWRQHSGQIH TLGGNTLYSM GAIGMCFNFG DYIAAGFKGD DSFI--LCHK YSEVKEYNTS
HHLV-1 MR644-18E/91	YEMGWFSLPA YKLMFTQRQS WKMSLIVDNQ TITSHSFLTG NFMQASGQPD TLGSNTYYDM AAVGMCID-I DFVAAFFKGD DSYI--IARR WKYAVFKGRK
TkHLV-2 MR233-17E/949	YEMGWFSLPA YKLMFTQRQS WKMSLIVDNQ TITSHSFLTG NFMQASGQPD TLGSNTYYDM AAVGMCID-I DFVAAFFKGD DSYI--IARR WKYAVFKGRK
TkHLV-17 MR233-17E/11883	LKGCGISRKI IDFYVDKRSK WSMVNITGTP NETLSTVLNG QWKQHSGQPF TLDGNTMYNM MLIGICYKFT ELSFASFKGD DSYI--CATK ID--------
HHLV-4 MR644-18E/70	LRQLGFNEKV LGKYLEMREK WKLVSATDS- --SFVMVMCN -WMQSSGQAF TLAGNTQHNM IVMGAAYIYK NIVFAMFKGD DSLI--VCEG IREAKIEGVL
MN823677 Forsythia suspensa hepe-like virus pt110-tom-3-NW	LKMMGFDGDL VNFYMAKRKD WTLINQDENF NMPLRTMLDG EYMQHSGQPF TLDGNTMFNM SCMGMCFEFV NMIFASFKGD DSYI--LAES IKPIMVGHDT
MK580123 Crustacea hepe-like virus 1 20180603	LRMMGCNQKI LGYYLKLRSQ WTLSSVSNDG PDNISMFLDG VWKQHSGQPF TLDGNTMFNM MAIGMCYDWT YLDAATFKGD DSAL--IGEG FKERIHDIRT
MF189998 Barns Ness breadcrumb sponge hepe-like virus 1 X15	FGLLGVNAKI INFYSSMRTK WCNLYQCREG ----ITMLHG LYMQHSGQPL TICGNTILNM AVLGYAYRIE NMLYASFKGD DSTI--RAKK ISTVPGKKTA
MK561971 Cragig virus 10	FGLLGVNHKI IDFYAKMRTE WCNLYQCNQG ----ISMLNG LYMQHSGQPL TITGNTILNM AVLGYAYRFQ EMLYAAFKGD DSHI--RAKK VSVVQGRKTA
TkHLV-1 MR233-17E/398	LTDIGVTDEC RQLYYEQRMD WTTVYPG--- ----FMVLKG KMKKQSGEIF TIDFNTAVCM AANGYMLDIV DLVLAQFKGD DSNI--AAED IR-LSKEGRE
HHLV-2_MR2018E/36	LTDIGVTDEC RQLYYEQRMD WTTVYPG--- ----FMVLKG KMKKQSGEIF TIDFNTAVCM AANGYMLDIV DLVLAQFKGD DSNI--AAED IR-LSKEGRE
HHLV-10 MR644-18E/1018	LIKIGVPHFL LEAFMDQLRN RRICCE---- ----VLSLIV NDKKDSGAPH TLIDNCLFNL AICLDLMVDF LYLY--IKGD DSFA--RGPK VRFDHERMSR
HHLV-6 MR644-18E/449	LVRIGCPEIL LEAFIEQITS RRICCE---- ----QSSLNV NDKKDSGAPH TLIDNCLFNL AICLDLMTDF EHLY--IKGD DSLA--RGRN VRF?HSRMND
MH188020 Culex bastrovirus-like virus CAVL/Fresno	LIRIGCPAKL VSYFCEQLKT RPLSA?---- ----QSSLVV NDKKDSGAPH TLVDNCLFNL AICLDVMTDF DKLY--IKGD DSLA--RGPN VAFNMERLNR
KX907135 Bastrovirus-like virus VietNam/Bat/17819_21	LLRIGAPPIL VSYFIEQLKT RRICAA---- ----QSSLVV NDKKDSGAPH TLVDNCLFNL AICLDVMQDF DKLY--IKGD DSLA--RGPD VSFNLPRLNH
TkHLV-31 MR233-17E/5834	LLRIGCPQLL VSYFIEQLKT RRICAA---- ----QSSLVV NDKKDSGAPH TLVDNCLFNL AICLDVMQDF DKLY--IKGD DSLA--RGRN VSFNLSRLND
MT568535 Pyongtaek Culex bastrovirus A18.1505	LLRIGCPEEL VELFCMQLSE RSVVDV---- ----FISVCA YDKKDSGAPH TLVDNCLFNL AVILDVLSDF RVLL--IKGD DSIA--FGEN IRFNKERASW
TkHLV-30 MR233-17E/17006	LAALGCPIQL RNNFLAMMRL RNVQSR---- ----VGTVQV QNKKDSGRVD TLIGNTLFNA AILLSCIDRT QIQYALMKGD DGLI--IGKA VRPNYKRIEE
KU318315 Bastrovirus 1	LKRLGLDPAV LDLYRDYRLS AKYSGPG--- ----ADIPAA -GMRMSGEAN TLLGNTMVTG ILHCAYVNQE PAWMM-FKGD DAIM--AGPD SMNVDVDS--
KU318318 Bastrovirus 2	LKRLGLDPAI LDLYRDYRLA AKYSGPG--- ----ADIPAA -GMRMSGEAN TLLGNTLVTG ILHCAYVNQE PAWMM-FKGD DAIM--AGPD SMDVDVET--
KU318317 Bastrovirus 3	LKRVGIDTQI AELYTEFRKN ATYSGPG--- ----ADLAAD -GMRMSGEAN TLLGNTLVTG ILHCAYLNQE PAWMM-FKGD DAII--AGPP EMELDCET--
MK387176 Porcine bastrovirus PBastV/USA/2017-1	LRTCGVPSSI CNAYRALREH AWVTAVD--- ----HAFSAD -HARMSGEAN TLFGNTAISL AVSSLLLPGE WACAA-FKGD DSFV--QSDC APLATDK---
MG693175 Bat bastrovirus CMR/Bat/P24	LRDLGVPDTI VRTYRALRES ARVSAQG--- ----VEAEAS -YARMSGEAN TLFGNTVVTM AVNALLFPGR FDWAA-FKGD DSII--CNPQ NRSDVDI---
MT549857 Bastrovirus CHN/Rat/78	LRWLGVPDSV CQLYLALRAA ARIESTM--- ----LCVDAP -NARMSGEAN TLFGNTIVTM AVNALLLPGS FSWAA-FKGD DSIV--AGPE HEAPVGG---
AY594352, *Omegatetravirus*, Dendrolimus punctatus tetravirus	LARVGTPAAA VTLFRQRCGK RTLRAKG--- ----IGSVEV DGLLDSGAVW TLTRNTTFSA GVMLTLFRGV KFAA--FKGD DSLL-VGSGP LTFTPARLHM
U18246, *Omegatetravirus*, Helicoverpa armigera stunt virus	LERIGTPAAA VNLFRERCGK RTLRAKG--- ----LGSVEV DGLLDSGAAW TPCRNTIFSA AVMLTLFRGV KFAA--FKGD DSLL-CGSHY LRFDASRLHM
AF102884, *Betatetravirus*, Nudaurelia capensis beta virus	CDR-GCPEHL IEYFEKRSKS RTLSSR---- ----IGSVDV SFMLDSGAVW TIARNTLFAS GLMLALFVGV DFIA--AKGD DVFL-AGNN- LYLDAERLRM
M73218, *Paslahepevirus*, Hepatitis E virus Burma	MEECGMPQWL IRLYHLIRSA WILQAP---- ----KESLRG FWKKHSGEPG TLLWNTVWNM AVITHCYDFR DFQVAAFKGD DSIV--LCSE YR-QSPGAAV
GU345042, *Rocahepevirus*, Hepatitis E virus rat/R63/DEU/2009	MKEAGAPEWM WRLYHLLRSG WVLQAP---- ----QESLRG RWKKHSGEPG TLLWNTVWNM AVIMHCYRFD GLAVAAFKGD DSIV--CCRF YQ-QEPRAAA
AY535004, *Avihepevirus*, Avian hepatitis E virus F93-5077	MRRFGMPDWM VALYHLVRSY WLLVAP---- ----KEALRG CWKKHSGEPG TLLWNTVWNM TVLHHVYEFD RPSVLCFKGD DSVV--VCES VR-ARPEGVS
JQ001749, *Chirohepevirus*, Bat hepevirus BatHEV/BS7/GE/2009	LTETGMPEWM VRLYWLQRAY WNLVAP---- ----DACLRG CWKKHSGEPG TLLFNTVWNM VVVNACYVFT DAVLHVYKGD DSVV--LCRS FE-EQPDAAH
HQ731075, *Piscihepevirus*, Cutthroat trout virus Heenan88	LVQCGMPPAL AEVYFNLRLK WTLDAR---- ----VASLRG TSKKHSGEPG TMLFNTIWNM TAVFHTTSFT RMTLALFKGD DSLV--CCDS SI-LTPTAKE
M15240, *Rubivirus*, Rubella virus F-therin	SAAL-LGLPC AEDYRALRAG SYCTLRE--- ----LGSTET GCERTSGEPA TLLHNTTVAM CMAMRMVPKG VRWAGIFQGD DMVIFLPEGA RSAALKWTPA
MN547623, *Rubivirus*, Ruhugu virus Cyclops leaf-nosed bat/2017/Uganda	SASL-LGLPS IEDYKALRAG SYCLLRD--- ----IASTET GCERTSGEPA TLLHNTLVGM CMAMRMVPKG VRWAGIFQGD DMVMFLPSQA LRTSLRWTPA
MN552442, *Rubivirus*, Rustrela virus Donkey/19_041-1/2019/Germany	SASL-LGLPS AEDYRALRSG SYCLIRD--- ----VAHTET GCERTSGEPA TLLHNTLVAT VMAMRMCSRC RGWAGVFQGD DMVLFFRDPG DRDALRWDCA
TkHLV-22 MR233-17E/663	MINMGFPKII VAKYWNAYVH WCQKARDD-- ---MEVTVDN VFMQHSGSPD TIHGNTKLTM GANGACYNFC TDGIIKRKKI EDVT---KAD CSAWLELNTD
HHLV-8 MR644-18E/220	MWRYGMPEVL VSYYYNFMET NTIKSLGK-- ----ILEMTL HFCRHSGGME TLWGNTNHSM NMIGLVMDFD DEGDWR---- ---------T AKFSESGMRI
TkHLV-32 MR233-17E/3264	MWKYGMPEVL VSYYYNFMER NEIKSLGK-- ----VLEMVM HFGRHSGGME TLWGNTNHSM NMIALVMDFD DEGDWR---- ---------T ARFSDAGMRI
MZ209973 Sanya hepevirus 1 TSYSY114	MGLYGMPAKL LRHYFYFSAN NHLKTMCG-- ----VLDMSL HFARHSGGYE TLWGNTNYSM ALVSMALELD HHTEWR---- ---------T ANVSERGLGF
TkHLV-33 MR233-17E/476	FRNHGMPQQL LDYLADKTRS YNIIDKSL-- ---IINLYGI MYGNCSGRFS TLFTNSDVTL SSVSQMYAWR KDADGQ---- -------VCG VTARGDLRSA
KX883762 Changjiang hepe-like virus 1 CJLX30636	YREFGMPEYL VQYNQKHAMK YSIMDTSR-- ---LLQLINV TCGNCSGKVQ TLPLNSAISL LICAYALDWR -EVDGT---- -------VLG FEKNARARAL
KX883749 Beihai barnacle virus 3 BHTH16145	LRRLGMPAFI IDSIWKMRER HSVRATDG-- ---TILMKNL QFGRDSGEPF TLDGNSLVTI AINIRALQPT VQECEL---- --------VD INPTSESFEA
KX883748 Beihai hepe-like virus 11 BHTH16134	LVDAGMPQHK LDLAMKLQRC YNIRTTDG-- ---SLTAKAV QCGRVSGQYR TFSGNSIVCM VGAAAIFSIT EAHGAP---- --------VG LECTNESIAQ
MW897133 Beijing sediment hepe-like virus 1165b-k141_200769	YREFGMPEYL IAYNQKHAMK YSIMDTSR-- ---LIQLINV TCGNCSGKVQ TLPLNSAISL LICAYTLDWK -EAGGE---- -------VLG FEKNARSRAL
MW897139 Sichuan sediment hepe-like virus 2 R45-k141_489063	YASYGMPEHL ILHNKEHAMK YNLLDSTG-- ---FLQMLNI LCGNCSGKPQ TLSLNSFVSM CILQNSLSWV TDSQNM---- -------PCD FSVRSDLRVA
MW897138 Xinjiang hepe-like virus 3 184-k141_405286	YRAYGMPEHL IRHNFEHANR YTIRTDTK-- ---AISMINV LAGNCSGKVQ TLPLNSAISM AICAYCVAWD VDASGN---- -------VYG FAPSARNRTA

Supplementary Figure 5 (continued)

TkHLV-4 MR233-17E/341	TSTYLGHKLK AE-MNF-FS- ---EFIANFI TPIG----FF PDVIRRASRV ISKVYT-HPD DWNKIRISVA DSLMVITNDN Q-
TkHLV-34 MR233-17E/510	LMQWCQYILK DS-VDV-VP- ---EFIANFL NPWG----FF PDVLRRVSRV VGRIVT-HPE QWEEMRRSVA DCLDVINNNS E-
MW346739 Hepeviridae sp. 1aq-RDRP-6	LWKLLGYKFK VS-FLN-YP- ---EYIANII TPEG----FF PDLVRRASRV ISKIYN-DKV DWAELRRSTA DSLAVINNGY S-
HHLV-3 MR644-18//1928	LWKHLGYQFK VN-LVK-YP- ---EYIANII TPEG----FF PDLIRRTSRV VSKIYG-DRM DWEETRMSTA DSLSVINNAY S-
HHLV-9 MR644-18E/14400	IYKRAGFDFK IE-TPV-IP- ---EYIANIV TPFG----FV PDLLRRVSRV VSKVYQ-TKA DWDQIKLSTA DALAVTPQAN M-
HHLV-1 MR644-18E/91	VVDIMGYQFK ID-TPE-IG- ---EFIANIV TPIG----FV PDYLRRVSRV VSKIYE-TNS DWNEIRLSTA DALSVVQQNN L-
TkHLV-2 MR233-17E/949	VVDIMGYQFK ID-TPE-IG- ---EFIANIV ---------- ---------- ---------- ---------- ---------- --
TkHLV-17 MR233-17E/11883	-ERLYGEK-K VR-------- ---DFCGYKI KDFR-----V PI-------- ---------- ---------- ---------- --
HHLV-4 MR644-18E/70	FTEHVGYKIK IH-EVD-IA- ---EYIANIV TPHG----WF PDVIRRVTRV LTKIYT-TKD DWLEIRKSMS DALDVID-PE NR
MN823677 Forsythia suspensa hepe-like virus pt110-tom-3-NW	LYAILSYKLK YD-FQN-IS- ---EYIANII TPTG----FF PDVLRRTSRI LTKIYT-VAD DWEEIKKSTA DALDVIADDQ E-
MK580123 Crustacea hepe-like virus 1 20180603	YIEITGYKIK AF-YVP-IL- ---EYISNIV TPAGK---FF PDVIRRVSRV VSKIYT-TQT DWEEQKLSIT DSLDVINTPE D-
MF189998 Barns Ness breadcrumb sponge hepe-like virus 1 X15	IYADHGYKLK IS-FEK-VS- ---EFIANFI TPFG----FF PDVVRRAVKA VSKVYE-DEE SWEESRINLK EVLSMVNSAE K-
MK561971 Cragig virus 10	IYVEHGYKLK IS-FEK-VS- ---EFIANFV TPYG----FF PDVVRRSVKA VSKVYE-DEE SWEESRVNLK EVLSMVNTAD K-
TkHLV-1 MR233-17E/398	FLINGGMKPK LH-IHEGSG- ---EFTGYIQ TPYG----PY PDTIRRVAKA FSKVFK-DEK DLDEFKLAMH DYVSGINNHA Q-
HHLV-2_MR2018E/36	FLINGGMKPK LH-IHEGSG- ---EFTGYIQ TPYG----PY PDTIRRVAKA FSKVFK-DEK DLDEFKLAMH DYVSGINNHA Q-
HHLV-10 MR644-18E/1018	YLKNCGYKFK PN-QHT-SG- ---SFVSFLV NQQG----VA LDLPRMCAKV TSRAYT-NVE DFLNYQEAIS AQLKNIDMQA G-
HHLV-6 MR644-18E/449	YI?KCG?VFK PE-KHT-SG- ---SFVSFLI NQNG----VA LDLPRICAKV TSRAYT-NIG DYHNYCDA?A GTLKTVDMQA G-
MH188020 Culex bastrovirus-like virus CAVL/Fresno	YNKQCGFQFK PN-SSA-VG- ---QFVSFLV SPRG----VA LDLARIAAKI TSRAYN-NKE DYDNYASALA GTLKPIDIDA G-
KX907135 Bastrovirus-like virus VietNam/Bat/17819_21	YNKTCGFQFK PN-TSS-VG- ---QFVSFLV SPRG----VA LDLARIAAKV TSRAYT-NKE DYDNYALALA GTLKPIDVDA G-
TkHLV-31 MR233-17E/5834	YNKKCGFQFK PN-SSS-VG- ---QFVSFLV SPRG----VA LDLARIAAKV TSRAYT-NKE DYDNYALALA GTLKPIDVDA G-
MT568535 Pyongtaek Culex bastrovirus A18.1505	YTKNCNYQFK PD-VSN-SG- ---EFVSFII NSEG----AS YDLPRLSSKV FSRVYT-SPQ DFRDYRDAVG VTLAHNPLTA G-
TkHLV-30 MR233-17E/17006	LEKNCSYKLK LT-VGK-SA- ---EFTSFII NGNG----AA LNIPRIAAKV LTRNYT--AD KYADYVVAVG DLIKSSN-NV G-
KU318315 Bastrovirus 1	MHKDYGMKAK LE-RME-VP- ---EFVSFFV SPYG----LL PDFFRIANKM VSNDHQDTPE WREASAANAR AVLELADNPA --
KU318318 Bastrovirus 2	MHKDYGMKAK LE-RME-VP- ---EFVSFFI SPYG----LL PDFFRIANKM CSNDHQDTPE WREASAANAR AILELTDNPA --
KU318317 Bastrovirus 3	MQEDYGMKAK PE-RME-VP- ---EFVSFFV SPYG----LL PDFFRIANKM VSNDHQPSPE WREASAANAR AILRMVTNPA --
MK387176 Porcine bastrovirus PBastV/USA/2017-1	IEDNIGMICK LE-RQE-VP- ---EFVSFLV A-PH----FA PDLKRCVGKL LGKPFPKE-- -VEELQQAVE NKLKLIHDPE --
MG693175 Bat bastrovirus CMR/Bat/P24	ISMQTGMQCK IE-ENH-VA- ---EFVGLLV SSDH----VF PDLRRRVGKL TGRGYPGDPL ARDQFAQSVK DTLALIPPAE --
MT549857 Bastrovirus CHN/Rat/78	VEQLTGMICK VE-KPE-VP- ---EFVGLVI S-DR----AY PDLRRAVGKL LGKGYPGDPI ARADLMRSVN ERLWLIPKDR --
AY594352, *Omegatetravirus*, Dendrolimus punctatus tetravirus	GEHYAKAHLK VE-TEV-VV- ---PYIGLLV SAEQ----VV PDPVRVALKV FGRCYS-THT LYGKYVDAVA DLVSSWADAA N-
U18246, *Omegatetravirus*, Helicoverpa armigera stunt virus	GERYKTKHLK VE-VQK-IV- ---PYIGLLV SAEQ----VV LDPVRSALKI FGRCYT-SEL LYSKYVEAVR DITKGWSDAR Y-
AF102884, *Betatetravirus*, Nudaurelia capensis beta virus	GSYLAANNLK IE-KTA-VV- ---SFIGFIV SQAA----VT ADVVRLATRT YGRSYK-NAD DLAKYKIAIA DHCKLFRSPR T-
M73218, *Paslahepevirus*, Hepatitis E virus Burma	LIAGCGLKLK VD-FRP-IG- ---LYAGVVV APGLG---AL PDVVRFAGRL TEKNWGPGPE RAEQLRLAVS DFLRKLTNVA Q-
GU345042, *Rocahepevirus*, Hepatitis E virus rat/R63/DEU/2009	LITGCGLKLK VN-FSD-VG- ---SYAGLLV ACGLG---VT PDVVRFLGRL SEKNWGPVKE RKEDLEQSVR DFVARLRNVT A-
AY535004, *Avihepevirus*, Avian hepatitis E virus F93-5077	LVADCGLKMK DK-TGP-CG- ---AFSNLLI FPGAG---VV CDLLRQWGRL TDKNWGPDIQ RMQDLEQACK DFVARVVTQG K-
JQ001749, *Chirohepevirus*, Bat hepevirus BatHEV/BS7/GE/2009	LITSCGLKLK VQ-FAG-IG- ---VFSHYIV APGEG---VV KDLLRTWGRM TEKNFS-DSE RSHDLCVAAQ DFVNSVTSQG K-
HQ731075, *Piscihepevirus*, Cutthroat trout virus Heenan88	FISQNGLVMK QK-TSH-LG- ---SFTCSLV TR-TS---YV PDVIRFACRL NNKDFT-DTK RIEDLQRATD DFIKRATVTT N-
M15240, *Rubivirus*, Rubella virus F-therin	EVGLFGFHIP VKHVSTPTP- ---SFCGHVG TAAG----LF HDVMHQAIKV LCRRFD--PD VLEEQQVALL DRLRGVYAAL PD
MN547623, *Rubivirus*, Ruhugu virus Cyclops leaf-nosed bat/2017/Uganda	ELGLYGFKIP VKHVATATP- ---SFCGHVG SADG----LF ADVLHLAIKL LCRKWD--PE VLGEQQTAML DRLMTVYAAL PA
MN552442, *Rubivirus*, Rustrela virus Donkey/19_041-1/2019/Germany	ELDLPGFVIP IKRVATATP- ---SFCGQLG SEAG----LF VDVLHLAVKL LCRRVD--PA LETERQEAML DKMKRVYGAL PD
TkHLV-22 MR233-17E/663	LQNAFGFRLK ED-FGN-PA- ---EFICNFI TDYG----FF PDVVRRSARY ISKVYQ-SVE DFQLTRDNIR DAVSTVTSDA --
HHLV-8 MR644-18E/220	MKAALGASLK MH-QGP-IG- ---DFVGYII QDFS----LR PDIFRLCAKV TSRKFALGTS VPNGLKAVYE QEYPALRN-- --
TkHLV-32 MR233-17E/3264	MKAALGAALK VH-EGP-IG- ---DFVGYII QDFS----LR PDIFRLSAKV ISRKFSLGTS VSAGLRAAYE ADYPELKS-- --
MZ209973 Sanya hepevirus 1 TSYSY114	LRKVFGAQLK LH-EGE-IA- ---DFVGFLI KDWR----AY PDIFRLSAKL LNRRFVDRCD VPKQTAGSYV SAHPKIGR-- --
TkHLV-33 MR233-17E/476	VEKLFGFEVK FG-AGE-IG- ---DFAGNVF ANGK----VY TDVMRMTAKT MCRRFL-LNR ALSKEQLAEM VADGYTPAE- --
KX883762 Changjiang hepe-like virus 1 CJLX30636	AEDMFGVQVK TG-AGV-VG- ---SFVGYLL HEGA----VV PDLLRMTGKF LDRQIY-SEA NIPKLDKMTL EEEGYKEHE- --
KX883749 Beihai barnacle virus 3 BHTH16145	AQDLFDMVIK HEFTHN-IG- ---EFIGYIF VAEANIPKFV PNIVKRVTKE LGRAYK--PQ DVKSQNKLVL QELREMGVHG --
KX883748 Beihai hepe-like virus 11 BHTH16134	AEKLLCMEYK YHRYNHVAGG AAADFIGFIA HDLAGTVRFF PDLPKRAAKE LTRKYV---- PFEDN--PQP EDLK------ --
MW897133 Beijing sediment hepe-like virus 1165b-k141_200769	AEEMFGVQVK TG-AGA-IG- ---SFVGYLV HEGA----VV PDLLRMTGKF LERQYF-SEA NVPKIDRLEL ENQGFRDNE- --
MW897139 Sichuan sediment hepe-like virus 2 R45-k141_489063	TEELFGVKIK VE-HGE-VA- ---SFIGYLI KD-------- ---------- ---------- ---------- ---------- --
MW897138 Xinjiang hepe-like virus 3 184-k141_405286	VEELFGVQIK HH-SGK-IG- ---SFVGYLL HAGR----MV PDLFRITGKF MARRLF-ANR AIDAAAELEM KALGYRGAD- --

Supplementary Table 1: Virus strains

Virus	GenBank accession number	Strain designation	Genome size	Coverage	Included in phylogenetic analyses of			Presence of contigs with >95% nt identity		Biological Sample (NCBI SRA)
					VMethylTr	Helicase	RdRp	Havel river sample	Teltowkanal samples	
**A. Havel hepe-like viruses**										
HHLV-1	OP699055	MR644-18E/91	8936 nt (cg)	43.9	+	+	+		TkHLV-2	MR644-18 (SRR21869516)
HHLV-2	OP699056	MR644-18E/36	8932 nt (cg)	18.0	+	+	+		TkHLV-1	MR644-18 (SRR21869516)
HHLV-3	OP699057	MR644-18E/70	9102 nt (cg)	13.1	+	+	+		TkHLV-16	MR644-18 (SRR21869516)
HHLV-4	OP699058	MR644-18E/1928	7902 nt (cg)	73.6	+	+	+		+	MR644-18 (SRR21869516)
HHLV-5	OP699059	MR644-18E/7839	7612 nt (cg)	20.9	+	+	+		TkHLV-20	MR644-18 (SRR21869516)
HHLV-6	OP699060	MR644-18E/449	5661 nt (cg)	13.0	+	+	+		TkHLV-28	MR644-18 (SRR21869516)
HHLV-7	OP699061	MR644-18E/184	4894 nt (par)	14.6	+	+	n.i.		-	MR644-18 (SRR21869516)
HHLV-8	OP699062	MR644-18E/220	4478 nt (par)	13.3	+	+	+		-	MR644-18 (SRR21869516)
HHLV-9	OP699063	MR644-18D/14400	1780 nt (par)	9.2	n.i.	n.i.	+		-	MR644-18 (SRR21869516)
HHLV-10	OP699064	MR644-18E/1018	2088 nt (par)	7.7	n.i.	n.i.	+		+	MR644-18 (SRR21869516)
HHLV-11	OP699065	MR644-18E/3095	1287 nt (par)	7.8	n.i.	n.i.	n.i.		-	MR644-18 (SRR21869516)
HHLV-12	OP699066	MR644-18E/3665	1204 nt (par)	8.0	n.i.	n.i.	n.i.		-	MR644-18 (SRR21869516)
HHLV-13	OP699067	MR644-18E/1769	1608 nt (par)	5.6	n.i.	+	n.i.		-	MR644-18 (SRR21869516)
HHLV-14	OP699068	MR644-18E/1089	5064 nt (par)	15.1	n.i.	n.i.	+		-	MR644-18 (SRR21869516)
HHLV-15	OP699069	MR644-18E/1511	1724 nt (par)	8.5	n.i.	+	+		-	MR644-18 (SRR21869516)
HHLV-16	OP699070	MR644-18E/3953	1375 nt (par)	4.4	+	n.i.	n.i.		+	MR644-18 (SRR21869516)
HHLV-17	OP699071	MR644-18E/1353	1819 nt (par)	47.2	n.i.	n.i.	n.i.		-	MR644-18 (SRR21869516)
HHLV-18	OP699072	MR644-18E/835	2309 nt (par)	14.7	n.i.	n.i.	n.i.		-	MR644-18 (SRR21869516)
HHLV-19	OP699073	MR644-18E/111514	2141 nt (par)	15.3	n.i.	+	+		-	MR644-18 (SRR21869516)
HHLV-20	OP699074	MR644-18E/1277	1874 nt (par)	7.4	n.i.	n.i.	n.i.		-	MR644-18 (SRR21869516)
HHLV-21	OP699075	MR644-18E/2015	1526 nt (par)	10.5	n.i.	+	n.i.		TkHLV-45	MR644-18 (SRR21869516)
HHLV-22	OP699076	MR644-18E/2223	1468 nt (par)	14.7	n.i.	n.i.	n.i.		-	MR644-18 (SRR21869516)
HHLV-23	OP699077	MR644-18E/31806	1541 nt (par)	3.1	n.i.	n.i.	n.i.		-	MR644-18 (SRR21869516)
HHLV-24	OP699078	MR644-18E/981	2125 nt (par)	5.6	+	n.i.	n.i.		+	MR644-18 (SRR21869516)
HHLV-25	OP699079	MR644-18E/1537	1709 nt (par)	9.7	+	n.i.	n.i.		+	MR644-18 (SRR21869516)
HHLV-26	OP699080	MR644-18E/2258	1459 nt (par)	6.6	+	n.i.	n.i.		-	MR644-18 (SRR21869516)
HHLV-27	OP699081	MR644-18E/3029	1300 nt (par)	4.9	n.i.	n.i.	n.i.		TkHLV-48	MR644-18 (SRR21869516)
HHLV-28	OP699082	MR644-18E/3455	1231 nt (par)	6.7	n.i.	n.i.	n.i.		-	MR644-18 (SRR21869516)
HHLV-29	OP699083	MR644-18E/5050	1067 nt (par)	7.4	n.i.	n.i.	n.i.		-	MR644-18 (SRR21869516)
HHLV-30	OP699084	MR644-18E/5051	1067 nt (par)	8.0	n.i.	n.i.	n.i.		-	MR644-18 (SRR21869516)
HHLV-31	OP699085	MR644-18E/5834	1006 nt (par)	5.1	n.i.	+	+		-	MR644-18 (SRR21869516)
HHLV-32	OP699086	MR644-18E/1558	1702 nt (par)	12.1	n.i.	+	+		-	MR644-18 (SRR21869516)
HHLV-33	OP699087	MR644-18E/6209	980 nt (par)	6.8	+	n.i.	n.i.		-	MR644-18 (SRR21869516)
HHLV-34	OP699088	MR644-18E/3095	1287 nt (par)	6.1	n.i.	n.i.	n.i.		-	MR644-18 (SRR21869516)
HHLV-35	OP699089	MR644-18E/4892	1080 nt (par)	7.3	n.i.	n.i.	n.i.		-	MR644-18 (SRR21869516)
HHLV-36	OP699090	MR644-18E/6360	970 nt (par)	7.5	n.i.	n.i.	n.i.			MR644-18 (SRR21869516)
HHLV-37	OP699091	MR644-18D/77135	1011 nt (par)	4.4	n.i.	n.i.	n.i.		TkHLV-33	MR644-18 (SRR21869516)
HHLV-38	OP699092	MR644-18E/10756	1964 nt (par)	7.4	n.i.	n.i.	n.i.		TkHLV-6	MR644-18 (SRR21869516)
**B. Teltowkanal hepe-like viruses**										
TkHLV-1	OP699093	MR233-17E/398	8934 nt (cg)	62.1	+	+	+	HHLV-2		MR233-17 (SRR21869517)
TkHLV-2	OP699094	MR233-17E/949	6377 nt (par)	13.6	+	+	+	HHLV-1		MR233-17 (SRR21869517)
TkHLV-3	OP699095	MR233-17E/415	8896 nt (cg)	76.9	+	+	+	+		MR233-17 (SRR21869517)
TkHLV-4	OP699096	MR233-17E/341	9208 nt (cg)	761.9	+	+	+	+		MR233-17 (SRR21869517)
	OP699097	20161205-D/3212	8995 nt (cg)	120.6	n.i.	n.i.	n.i.	-		20161205 (SRR21869518)
TkHLV-5	OP699098	MR233-17E/15319	1572 nt (par)	7.4	n.i.	n.i.	n.i.	-		MR233-17 (SRR21869517)
TkHLV-6	OP699099	MR233-17E/27572	2715 nt (par)	13.3	+	n.i.	n.i.	HHLV-38		MR233-17 (SRR21869517)
TkHLV-7	OP699100	MR233-17E/19201	1403 nt (par)	5.8	+	n.i.	n.i.	+		MR233-17 (SRR21869517)
TkHLV-8	OP699101	MR233-17E/430	8817 nt (cg)	566.0	+	+	+	+		MR233-17 (SRR21869517)
	OP699102	20161205-D/10029	8717 nt (par)	147.1	n.i.	n.i.	n.i.	-		20161205 (SRR21869518)
TkHLV-9	OP699103	MR233-17E/1114	5982 nt (par)	37.5	+	+	+	-		MR233-17 (SRR21869517)
TkHLV-10	OP699104	MR233-17E/49852	7452 nt (par)	15.4	+	+	+	-		MR233-17 (SRR21869517)
TkHLV-11	OP699105	MR233-17E/15966	1539 nt (par)	7.7	n.i.	n.i.	n.i.	-		MR233-17 (SRR21869517)
TkHLV-12	OP699106	20161205-D/2309	4574 nt (par)	414.9	n.i.	n.i.	n.i.	-		20161205 (SRR21869518)
	OP699107	MR233-17E/582	8061 nt (cg)	1987.1	+	+	+	+		MR233-17 (SRR21869517)
TkHLV-13	OP699108	MR233-17E/1063	6083 nt (par)	15.0	+	+	+	+		MR233-17 (SRR21869517)
TkHLV-14	OP699109	MR233-17E/18190	1442 nt (par)	8.8	n.i.	n.i.	n.i.	+		MR233-17 (SRR21869517)
TkHLV-15	OP699110	MR233-17E/10927	1864 nt (par)	25.3	n.i.	n.i.	n.i.	-		MR233-17 (SRR21869517)
TkHLV-16	OP699111	20161205-D/18546	6893 nt (par)	23.3	n.i.	n.i.	n.i.	HHLV-4		20161205 (SRR21869518)
	OP699112	MR233-17E/325	9268 nt (cg)	189.7	+	+	+	HHLV-4		MR233-17 (SRR21869517)
TkHLV-17	OP699113	MR233-17E/11883	1788 nt (par)	6.9	n.i.	n.i.	+	-		MR233-17 (SRR21869517)
TkHLV-18	OP699114	20161205-D/8739	9158 nt (cg)	129.1	n.i.	n.i.	n.i.	HHLV-27		20161205 (SRR21869518)
	OP699115	MR233-17E/349	9184 nt (cg)	439.9	+	+	+	HHLV-27		MR233-17 (SRR21869517)
TkHLV-19	OP699116	MR233-17E/20784	1348 nt (par)	5.34	n.i.	n.i.	n.i.	+		MR233-17 (SRR21869517)
TkHLV-20	OP699117	MR137-16D/77	7678 nt (cg)	681.8	n.i.	n.i.	n.i.	HHLV-5		MR137-16 (SRR21869519)
	OP699118	MR233-17E/643	7758 nt (cg)	4444.4	+	+	+	HHLV-5		MR233-17 (SRR21869517)
TkHLV-21	OP699119	MR233-17E/1352118	5461 nt (par)	63.0	+	+	+	-		MR233-17 (SRR21869517)
TkHLV-22	OP699120	MR233-17E/663	7653 nt (cg)	448.9	+	+	+	+		MR233-17 (SRR21869517)
TkHLV-23	OP699121	MR233-17E/14711	1602 nt (par)	8.2	n.i.	n.i.	n.i.	-		MR233-17 (SRR21869517)
TkHLV-24	OP699122	MR233-17E/708	7785 nt (par)	71.5	+	+	+	+		MR233-17 (SRR21869517)
TkHLV-25	OP699123	MR233-17E/401	8927 nt (cg)	37.9	+	+	+	-		MR233-17 (SRR21869517)
TkHLV-26	OP699124	MR233-17E/7023	2338 nt (par)	30.8	n.i.	n.i.	n.i.	-		MR233-17 (SRR21869517)
TkHLV-27	OP699125	MR233-17E/7149	2315 nt (par)	41.4	n.i.	n.i.	n.i.	-		MR233-17 (SRR21869517)
TkHLV-28	OP699126	MR233-17E/11756	5672 nt (par)	16.6	+	+	+	HHLV-6		MR233-17 (SRR21869517)
TkHLV-29	OP699127	MR233-17E/5607	2640 nt (par)	13.9	+	+	n.i.	+		MR233-17 (SRR21869517)
TkHLV-30	OP699128	MR233-17E/17006	2613 nt (par)	6.0	n.i.	n.i.	+	+		MR233-17 (SRR21869517)
TkHLV-31	OP699129	MR233-17E/26350	1200 nt (par)	14.5	n.i.	n.i.	+	-		MR233-17 (SRR21869517)
TkHLV-32	OP699130	MR233-17E/3264	9266 nt (cg)	18.5	+	+	+	+		MR233-17 (SRR21869517)
TkHLV-33	OP699131	MR233-17E/476	8587 nt (cg)	52.7	+	+	+	HHLV-37		MR233-17 (SRR21869517)
TkHLV-34	OP699132	MR233-17E/510	8452 nt (cg)	237.1	+	+	+	+		MR233-17 (SRR21869517)
	OP699133	MR137-16D/328	8220 nt (par)	243.6	n.i.	n.i.	n.i.	+		MR137-16 (SRR21869519)
	OP699134	20161205-D/3071	1203 nt (par)	21.8	n.i.	n.i.	n.i.	+		20161205 (SRR21869518)

TkHLV-35	OP699135	MR233-17E/33892	1062 nt (par)	5.3	n.i.	n.i.	n.i.	-		MR233-17 (SRR21869517)
TkHLV-36	OP699136	MR233-17E/595	8024 nt (cg)	1217.9	+	+	+	+		MR233-17 (SRR21869517)
	OP699137	20161205-D/4059	7940 nt (cg)	224.7	n.i.	n.i.	n.i.	+		20161205 (SRR21869518)
TkHLV-37	OP699138	MR233-17E/10440	1908 nt (par)	9.8	n.i.	+	n.i.	-		MR233-17 (SRR21869517)
TkHLV-38	OP699139	MR233-17E/6603	2417 nt (par)	7.2	+	+	n.i.	+		MR233-17 (SRR21869517)
TkHLV-39	OP699140	MR233-17E/503	8483 nt (cg)	371.4	+	+	+	+		MR233-17 (SRR21869517)
TkHLV-40	OP699141	MR233-17E/490	8541 nt (par)	81.5	+	+	+	+		MR233-17 (SRR21869517)
TkHLV-41	OP699142	MR233-17E/1088	6035 nt (par)	21.2	n.i.	+	+	-		MR233-17 (SRR21869517)
TkHLV-42	OP699143	MR233-17E/9232	2031 nt (par)	9.8	n.i.	n.i.	+	-		MR233-17 (SRR21869517)
TkHLV-43	OP699144	MR233-17E/10363	1915 nt (par)	9.4	n.i.	n.i.	n.i.	+		MR233-17 (SRR21869517)
TkHLV-44	OP699145	MR233-17E/406908	1458 nt (par)	14.1	+	n.i.	n.i.	-		MR233-17 (SRR21869517)
TkHLV-45	OP699146	MR233-17E/1231	5700 nt (par)	15.8	n.i.	+	+	HHLV-21		MR233-17 (SRR21869517)
TkHLV-46	OP699147	MR233-17E/14194	1631 nt (par)	9.5	n.i.	n.i.	n.i.	-		MR233-17 (SRR21869517)
**C. Astro-like viruses**										
HALV-1	OP699148	MR644-18E/601	3273 nt (par)	7.2	n.i.	n.i.	+		+	MR644-18 (SRR21869516)
HALV-2	OP699149	MR644-18E/1796	1589 nt (par)	7.1	n.i.	n.i.	+		+	MR644-18 (SRR21869516)
HALV-3	OP699150	MR644-18E/25367	539 nt (par)	2.6	n.i.	n.i.	+		-	MR644-18 (SRR21869516)
TkALV-1	OP699151	MR233-17E/955	6353 nt (par)	153.6	n.i.	n.i.	+	+		MR233-17 (SRR21869517)
TkALV-2	OP699152	MR233-17E/7172	2312 nt (par)	2.8	n.i.	n.i.	n.i.	-		MR233-17 (SRR21869517)
TkALV-3	OP699153	MR233-17E/832	6815 nt (par)	440.6	n.i.	n.i.	+	+		MR233-17 (SRR21869517)

Abbreviations: cg, almost complete genome; par, partial genome; n.i., not included; RdRp, RNA-dependent RNA polymerase; VMethylTr, viral methyltransferase
